# New insights into donor directionality of mating-type switching in *Schizosaccharomyces pombe*

**DOI:** 10.1371/journal.pgen.1007424

**Published:** 2018-05-31

**Authors:** Takahisa Maki, Naoto Ogura, James E. Haber, Hiroshi Iwasaki, Geneviève Thon

**Affiliations:** 1 Institute of Innovative Research, Tokyo Institute of Technology, Tokyo, Japan; 2 School of Life Science and Technology, Department of Life Science and Technology, Tokyo Institute of Technology, Tokyo, Japan; 3 Department of Biology and Rosenstiel Basic Medical Science Research Center, Brandeis University, Waltham, Massachusetts, United States of America; 4 Department of Biology, University of Copenhagen, BioCenter, Copenhagen, Denmark; University of Sussex, UNITED KINGDOM

## Abstract

Mating-type switching in *Schizosaccharomyces pombe* entails programmed gene conversion events regulated by DNA replication, heterochromatin, and the HP1-like chromodomain protein Swi6. The whole mechanism remains to be fully understood. Using a gene deletion library, we screened ~ 3400 mutants for defects in the donor selection step where a heterochromatic locus, *mat2-P* or *mat3-M*, is chosen to convert the expressed *mat1* locus. By measuring the biases in *mat1* content that result from faulty directionality, we identified in total 20 factors required for donor selection. Unexpectedly, these included the histone H3 lysine 4 (H3K4) methyltransferase complex subunits Set1, Swd1, Swd2, Swd3, Spf1 and Ash2, the BRE1-like ubiquitin ligase Brl2 and the Elongator complex subunit Elp6. The mutant defects were investigated in strains with reversed donor loci (*mat2-M mat3-P*) or when the *SRE2* and *SRE3* recombination enhancers, adjacent to the donors, were deleted or transposed. Mutants in Set1C, Brl2 or Elp6 altered balanced donor usage away from *mat2* and the *SRE2* enhancer, towards *mat3* and the *SRE3* enhancer. The defects in these mutants were qualitatively similar to heterochromatin mutants lacking Swi6, the NAD^+^-dependent histone deacetylase Sir2, or the Clr4, Raf1 or Rik1 subunits of the histone H3 lysine 9 (H3K9) methyltransferase complex, albeit not as extreme. Other mutants showed clonal biases in switching. This was the case for mutants in the NAD^+^-independent deacetylase complex subunits Clr1, Clr2 and Clr3, the casein kinase CK2 subunit Ckb1, the ubiquitin ligase component Pof3, and the CENP-B homologue Cbp1, as well as for double mutants lacking Swi6 and Brl2, Pof3, or Cbp1. Thus, we propose that Set1C cooperates with Swi6 and heterochromatin to direct donor choice to *mat2-P* in M cells, perhaps by inhibiting the *SRE3* recombination enhancer, and that in the absence of Swi6 other factors are still capable of imposing biases to donor choice.

## Author summary

Effects of chromatin structure on recombination can be studied in the fission yeast *S*. *pombe* where two heterochromatic loci, *mat2* and *mat3*, are chosen in a cell-type specific manner to convert the expressed *mat1* locus and switch the yeast mating-type. The system has previously revealed the determining role of heterochromatin, histone H3K9 methylation and HP1 family protein Swi6, in donor selection. Here, we find that other chromatin modifiers and protein complexes, including components of the histone H3K4 methyltransferase complex Set1C, the histone H2B ubiquitin ligase HULC and Elongator, also participate in donor selection. Our findings open up new research paths to study mating-type switching in fission yeast and the roles of these complexes in recombination.

## Introduction

**Fig 1:**
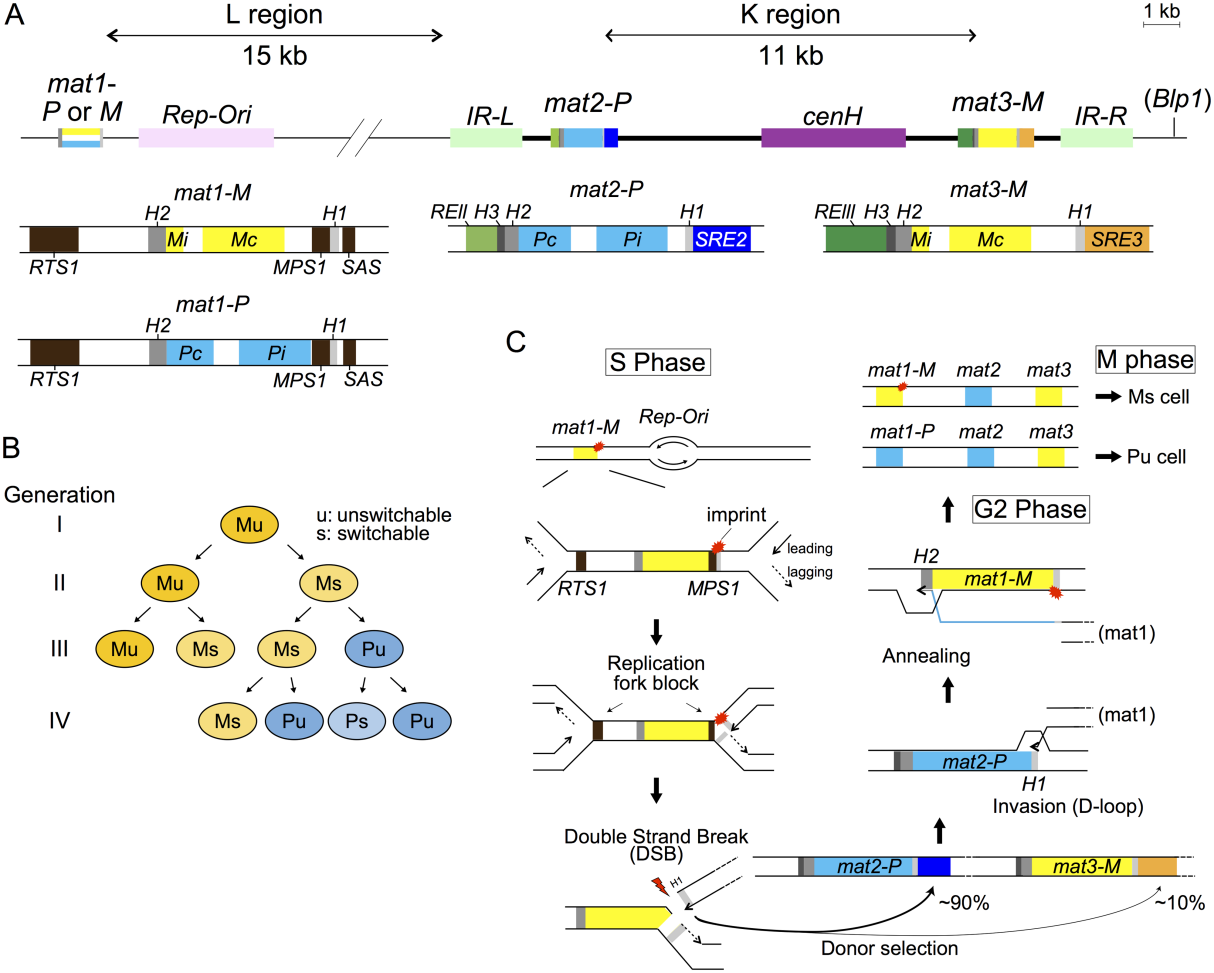
Mating-type switching in *S*. *pombe*. (A) The *mat1* locus is expressed and confers cell type. The silent loci *mat2-P* and *mat3-M* serve as donors of genetic information to interconvert the *mat1-P* and *mat1-M* alleles. They are located in a heterochromatic region bordered by the *IR-L* and *IR-R* repeats. The small *H1* and *H2* homology boxes are common to all cassettes. *H3* boxes are specific to *mat2-P* and *mat3-M*. *RTS1*: Replication Termination Site 1; *MPS1*: *mat1*-Pausing Site 1; *SAS*: Switch Activation Site; *IR-L* or *R*: Inverted Repeat Left or Right; *RE*: Repressor Element; *SRE*: Swi2-dependent Recombination Enhancer; *cenH*: centromere Homology; *Rep-Ori*: origin of replication. (B) Mating-type switching occurs in a defined pattern depicted here, with P cells represented in blue and M cells in yellow. The subscripts ‘u’ and ‘s’ designate unswitchable and switchable cells, respectively. (C) Steps in mating-type switching. A DNA modification or ‘imprint’ at the *mat1-H1* junction is symbolized by a red mark. Leading-strand synthesis incoming from the indicated origin of DNA replication proceeds through the *mat1-H1* homology box and stops at this modification creating a one-ended double-strand break (DSB). The resulting free DNA end invades the *H1* box of the silent cassette containing the information for the opposite mating-type, *mat2-P* in this example. D-loop formation permits extension of the DNA molecule at *mat2-P*, until the newly synthesized *H2* sequence copied from *mat2-P* anneals back to the *H2* box at *mat1* to resolve recombination. Completion of switching further requires second-strand synthesis (of *mat1-P* in the case shown) and degradation of the unused *mat1* template strand.

The fission yeast *S*. *pombe* exists as two haploid cell types, plus (*P*) and minus (*M*), that differ at the *mat1* locus. When starved for nitrogen, haploid cells undergo sexual differentiation, mate with the opposite cell type and sporulate. These events are driven by master regulators expressed from the *mat1-P* and *mat1-M* alleles [1]. The regulators first drive sexual differentiation and mating and, when co-expressed in the zygote, meiosis and sporulation. Homothallic (*h*^*90*^) colonies sporulate very efficiently because they contain equal proportions of P and M cells due to frequent gene conversions at *mat1*. The genetic information at *mat1* is replaced with genetic information copied from one of two silent loci, *mat2-P* or *mat3-M* [2]. The organization of the ~35 kb region of chromosome 2 that comprises *mat1*, *mat2-P* and *mat3-M* in *h*^*90*^ strains is depicted in Fig 1A. All three loci are flanked by short regions of sequence identity, the centromere-distal *H1* box and the centromere-proximal *H2* box. In addition, *H3* homology boxes immediately adjacent to *H2* are found exclusively at *mat2-P* and *mat3-M*. An alternative arrangement, known as *h*^*09*^, has *mat2-M* and *mat3-P* cassettes [3].

Mating-type switching follows the so-called ‘Miyata’s rules’ inferred from pedigree analyses of dividing cells [4]. A single *h*^*90*^ cell produces both ‘unswitchable’ and ‘switchable’ cells. According to the ‘one-in-four-rule’, illustrated in Fig 1B, one of two sister cells originating from an ‘unswitchable’ cell becomes ‘switchable’. That cell produces one switched daughter upon cell division and one unswitched, but switchable, daughter. Thus, only one cell out of four cousins displays a switched mating-type (the ‘one-in-four’ rule [4]) and lineages of ‘switchable’ cells are observed (the ‘recurrent switching’ rule [5]). The known mechanisms of mating-type switching provide an explanation for these rules.

Mating-type switching is initiated by an imprint at *mat1* introduced during DNA replication [6, 7]. Replication stalls within *mat1* at the *MPS1* site and a nick or two ribonucleotides are incorporated into the lagging strand at the imprinting site situated nearby, at the junction of the *mat1* cell-type specific information and *H1* box [6–15]. This imprint creates a ‘switchable’ cell. During the next round of DNA synthesis, the imprint is converted into a double-strand break (DSB) that triggers homologous recombination and mating-type switching [10, 14]. At least seven factors (Swi1, Swi3, Pol1 (Swi7), Sap1, Lsd1, Lsd2 and Mrc1) are required for efficient DSB formation. The Swi1-Swi3 complex and Mrc1 are necessary for imprinting by pausing replication forks at *MPS1* [7, 16]. The DNA primase Pol1 and a DNA element targeted by the essential DNA-binding protein Sap1 are not required for replication fork stalling at *MPS1*; hence Pol1 and Sap1 are believed to catalyze imprint formation downstream of the Swi1-Swi3 complex [7], while Lsd1 and Lsd2 are required upstream of Swi1-Swi3 [17, 18]. In addition, Swi1, Swi3 and Mrc1 block replication from the *mat1* distal side at the replication termination site *RTS1* to optimize mating-type switching [7, 16]. Thus, replication proceeds unidirectionally when leading-strand synthesis reaches the imprint and the nick is converted into a one-ended DSB.

The DSB end initiates repair by recombining with one of the heterochromatic and transcriptionally silent cassettes *mat2-P* and *mat3-M* [10, 19, 20]. The free DNA end can invade either *mat2-P* or *mat3-M*, however mating-type information opposite to the information present at *mat1* is chosen with a ~90% probability, leading to Miyata’s observation that switchable cells nearly always switch to the opposite mating-type [4, 9]. Surprisingly, this strongly biased donor selection relies on the heterochromatic state of the *mat2*-*mat3* region [3, 21–24]. In the wild-type, histone H3K9 methylation deposited between the inverted repeat boundaries *IR-L* and *IR-R* permits the binding of the key switching factor Swi6, an HP1 homolog [25]. Defective donor choice in *swi6* mutants biases *h*^*90*^ cell populations towards the M mating-type due to preferred use of a *mat3-M* adjacent recombination enhancer over a *mat2-P* adjacent enhancer in the absence of Swi6 [22, 23, 26]. Also essential at this step of switching is the Swi2-Swi5 complex, capable of interacting with Swi6, and whose molecular role is inferred from the related Sfr1-Swi5 complex [27, 28]. Sfr1 shares sequence homology with the C-terminus of Swi2, in a domain that permits the interaction of either Swi2 or Sfr1 with the recombination mediator Swi5 and with the strand-exchange factor Rad51. The Sfr1-Swi5 complex stabilizes Rad51 filaments *in vivo* and promotes Rad51-mediated strand exchange *in vitro* [28–30]. Consistent with similar functions for the Swi2-Swi5 complex, Swi2-Swi5 interacts with Rad51 in two-hybrid assays [27]. Thus, mechanistically, the ability of Swi2-Swi5 to interact with Swi6 suggests that the complex participates in donor choice by biasing strand invasion [27]. This idea is supported by cell-type specific associations of Swi2 and Swi5 with the mating-type region [22]. Swi2 localizes to the *mat2-P* and *mat3-M* adjacent enhancers *SRE2* and *SRE3* (Swi2-dependent recombination enhancer element 2 and 3) in M cells, but only to *SRE3* in P cells [22, 23]. In *swi6* mutant cells, Swi2 localizes only to *SRE3*, as in P cells [22]. These observations indicate that the heterochromatin-mediated localization of Swi6 regulates Swi2-Swi5 localization to *SRE2* to choose *mat2-P* [22, 23, 31].

After strand invasion into the homologous sequence at the *H1* homology box, the donor locus information is copied by polymerase extension until it reaches the *H3* homology box, then *H3* is believed to form a hairpin loop structure [32]. The mismatch repair Msh2 (Swi8)-Msh3 (Swi4) complex recognizes this conformation and DNA synthesis stops at the donor cassette. The Rad51 mediator Rad55-Rad57 has been suggested to work together with Msh2 (Swi8), because mutants in these factors tend to form *h*^*+N*^ rearrangements containing a duplication of the entire *mat2-*3 region at *mat1* [33–36]. In addition to Rad55-Rad57, the homologous recombination factor Rad52 is required for DSB repair at *mat1* [37, 38]. Presumably, Rad55-Rad57 and Rad52 are involved in annealing between two *H2* boxes in *mat1* and the donor cassette. The endonuclease Rad16 (Swi9)-Swi10 and its activator Pxd1 cleave the intermediate between the *H2* and *H3* boxes [37, 39]. This is followed by new DNA synthesis from *H2* of *mat1* to *H1* to complete replication of this region and to thus switch mating-type.

This switching system has been utilized to study multiple aspects of replication, histone modification and recombination. Historically, the mating-type switching related genes were classified functionally by Southern blotting analysis of the effect of mutants on *mat1* switching [40]. Class Ia genes (*swi1*, *3*, and *7*) are required for the imprinting step that leads to the DSB formation, hence mutants in these genes show no DSB in a Southern blot. Class Ib genes (*swi2*, *5* and *6*) are not necessary for DSB formation, but required for efficient switching. The third group, Class II, (*swi4*, *8*, *9*, and *10*) resolves the recombination intermediate, mutants in these genes contain frequent rearrangements of the mating-type region, in particular the *h*^*+N*^ duplication. Subsequent screens identified additional factors (Table 1) and suggested that yet more might exist. In particular, aspects of imprint formation and donor choice are still not understood.

**Table 1:** Mating-type switching related genes.

Systematic ID	Gene	Gene description	Ver5.0 position	reference
SPBC216.06c	swi1	replication fork protection complex subunit Swi1	V5-P36-57	[40]
SPAC1142.03c	swi2	Swi5 complex subunit Swi2	V5-P02-96	[40]
SPBC30D10.04	swi3	replication fork protection complex subunit Swi3	V5-P21-55	[40]
SPAC8F11.03	msh3 (swi4)	MutS protein homolog 3	V5-P05-64	[40]
SPBC409.03	swi5	Swi5 protein	V5-P30-21	[40]
SPAC664.01c	swi6	chromodomain protein Swi6	V5-P05-31	[40]
SPAC3H5.06c	pol1 (swi7)	DNA polymerase alpha catalytic subunit	-	[40]
SPBC19G7.01c	msh2 (swi8)	MutS protein homolog 2	V5-P31-52	[40]
SPCC970.01	rad16 (swi9)	DNA repair endonuclease XPF	V5-P23-76	[40]
SPBC4F6.15c	swi10	DNA repair endonuclease	V5-P34-76	[40]
SPCC1672.02c	sap1	switch-activating protein Sap1	-	[41]
SPAC30D11.10	Rad52	DNA repair protein Rad52	V5-P10-40	[38]
SPBC2D10.17	clr1	cryptic loci regulator Clr1	V5-P12-69	[42]
SPAC1B3.17	clr2	chromatin silencing protein Clr2	V5-P10-18	[43]
SPBC800.03	clr3	histone deacetylase (class II) Clr3	V5-P09-57	[43]
SPBC428.08c	clr4	histone H3 methyltransferase Clr4	V5-P12-80	[43]
SPCC11E10.08	rik1	silencing protein Rik1	V5-P08-03	[43]
SPAC18B11.07c	rhp6	Rad6 homolog Rhp6	V5-P03-66	[44]
SPAC17H9.20	psc3	mitotic cohesin complex, non-SMC subunit Psc3	-	[45]
SPBC16D10.07c	sir2	Sir2 family histone deacetylase Sir2	V5-P13-87	[46]
SPAC3A11.08	pcu4 (cul4)	cullin 4	V5-P36-16	[47]
SPCC613.12c	raf1 (clr8)	Rik1-associated factor Raf1	V5-P26-35	[24]
SPCC970.07c	raf2 (clr7)	Rik1-associated factor Raf2	V5-P27-50	[24]
SPAC3C7.03c	rad55	RecA family ATPase Rad55	V5-P13-53	[35]
SPAC20H4.07	rad57	RecA family ATPase Rad57	V5-P25-35	[36]
SPAC644.14c	rad51	recombinase Rsd51	V5-P05-30	[36]
SPAC15A10.03c	rad54	Rad54 homolog Rad54	V5-P11-89	[36]
SPAC1556.01c	rad50	DNA repair protein Rad50	V5-P23-44	[36]
SPCC4G3.07c	phf1	PHD finger containing protein Phf1	-	[36]
SPBC1105.04c	cbp1	CENP-B homolog	V5-P09-19	[48]
SPAC343.11c	msc1	multi-copy suppressor of Chk1	V5-P04-71	[49]
SPBC23G7.09	mat1-mc	Mating-type m-specific polypeptide Mc	-	[26]
SPBC106.09	cut4	platform subcomplex scaffold subunit Apc1	-	[50]
SPAC6F12.15c	cut9	TPR lobe subcomplex subunit Cut9/Apc6	-	[50]
SPBC146.09c	lsd1	histone demethylase SWIRM1	V5-P06-14	[17]
SPAC23E2.02	lsd2	histone demethylase SWIRM2	-	[17]
SPCC1322.02	pxd1	sequence orphan	V5-P11-47	[39]
SPAC694.06c	mrc1	mediator of replication checkpoint 1	V5-P05-35	[16]
SPBC3H7.10	elp6	elongator homolog	V5-P21-32	This study
SPCC970.10c	brl2	ubiquitin-protein ligase E3	V5-P08-48	This study
SPAC1851.03	ckb1	CK2 family regulatory subunit	V5-P03-65	This study
SPCC338.16	pof3	F-box protein Pof3	V5-P09-78	This study
SPAC23H3.05c	swd1	COMPASS complex subunit Swd1	V5-P13-45	This study
SPBC18H10.06c	swd2	COMPASS complex subunit Swd2	V5-P06-59	This study
SPCC594.05c	spf1	COMPASS complex subunit	V5-P28-74	This study
SPCC306.04c	set1	histone lysine methyltransferase Set1	V5-P27-78	This study
SPBC354.03	swd3	WD repeat protein Swd3	V5-P27-21	This study
SPBC13G1.08c	ash2	Ash2-trithorax family protein	V5-P10-78	This study

To identify yet unknown regulators of mating-type switching, we combined the deletion of 3420 nonessential genes (Bioneer gene deletion library version 5) with an *h*^*90*^ strain background. The strain also included a dual reporter system with CFP under the control of a *P*-specific promoter and YFP under the control of an *M*-specific promoter, so that the ratio of *P*-to-*M* cells was determined by comparing CFP and YFP fluorescence. As a secondary screening strategy, the genetic content at *mat1* was quantified with multiplex PCR using genomic DNA isolated from the candidates that passed the initial screen. These extensive screens identified several new mating-type switching genes whose deletion results in a bias toward *M* cells within *h*^*90*^ populations instead of the balanced P:M ratio. In addition, analysis of *h*^*09*^ strains revealed that some strains showed clonal biases in independent colonies. As mentioned above, Swi6 is an essential directionality factor [3, 22, 23]. Epistasis analysis with *swi6Δ* suggests that Clr4, Sir2, Swd1, Clr3 work in the same pathway as Swi6, whereas Brl2, Pof3 and Cbp1 act *via* Swi6-dependent and -independent mechanisms. These observations provide new clues to understand the molecular mechanisms of mating-type switching.

## Results and discussion

### Identification of mating-type switching defective mutants

**Fig 2:**
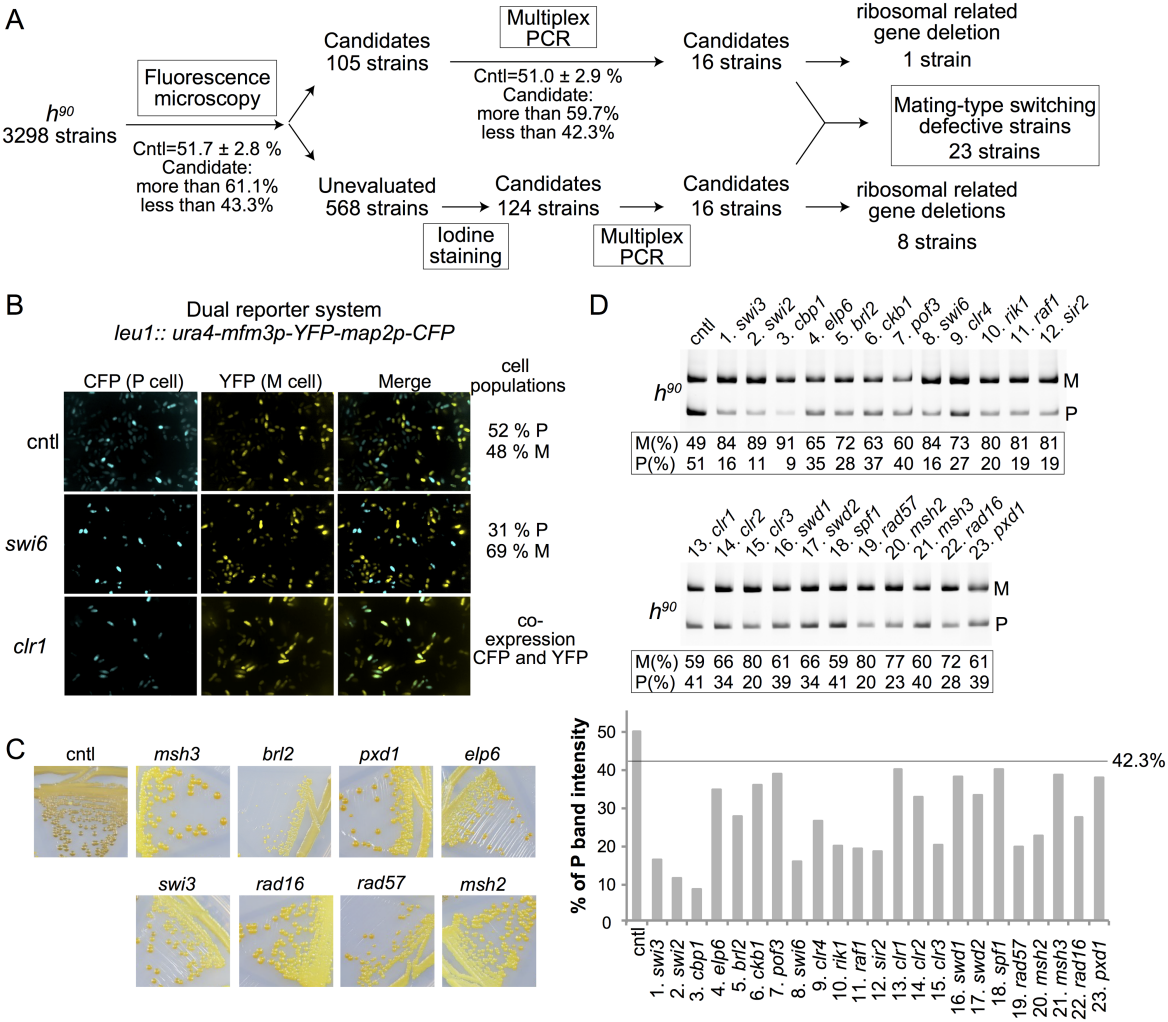
Screen for mutants defective in donor choice. (A) Summary of screen. Fluorescence microscopy, multiplex PCR for *mat1* content and iodine staining were used as described in the text to identify mutants with a *mat1* content biased towards *mat1-P* or *mat1-M*. Candidates were selected for differing by > 3 standard deviations from the mean in the fluorescence and multiplex PCR tests. Lists of mutants and values are presented in S2–S4 Tables. (B) Examples of cell-type ratios measured by fluorescence microscopy with the dual reporter system using YFP under control of the *mfm3* M-specific promoter and CFP under control of the *map2* P-specific promoter. P-to-M cell ratios were determined with an Opera high-throughput microscope (Perkin Elmer Inc.). % of cell population were calculated from cyan/(cyan + yellow) cell ratios. The unmutagenized strain PG4045 is shown as control (cntl). See S2 Fig for additional mutants. (C) Spore content was assayed by exposure of colonies to iodine vapor. The starch in spore walls is stained darkly by iodine. Mating-type switching mutants form light colonies. (D) Multiplex PCR was used to measure *mat1* content with a P- and an M-specific primer combined with a *mat1*-specific primer. The P and M band intensities in each lane were used to calculate P/(P+M) and M/(P+M) ratios reported under the gel pictures and as bar graphs for P/(P+M). The line at 42.3% in the graph shows the lower limit accepted as normal mating-type switching.

We conducted a genome-wide screen for factors required for mating-type switching. The screen used an *S*. *pombe* gene deletion library (Bioneer) consisting of 3420 haploid strains, each of which lacks a non-essential gene. Mating-type switching occurs in *h*^*90*^ strains, yet the Bioneer library strains are heterothallic *h*^*+N*^ strains for which a large duplication in the mating-type region abrogates mating-type switching. To construct *h*^*90*^ derivatives of the entire Bioneer collection, strain PG4045 was mated to the library. The *h*^*90*^ mating-type region of PG4045 could be selected in the progeny due to the linked *LEU2* gene. In addition, PG4045 contains two fluorescent reporters specific for the *P* (CFP controlled by the *map2* promoter) and *M* (YFP controlled by the *mfm3* promoter) cell types, respectively. Both reporters are expressed from the *leu1* locus where they were integrated together with the selectable *ura4*^+^ gene. Thus, we selected *h*^*90*^ *LEU2 ura4*^+^ segregants (S1 Fig). We obtained 3298 *h*^*90*^ deletion strains in which we monitored expression of the fluorescent reporters (Fig 2A). Efficient mating-type switching results in rapid homogenization of *h*^*90*^ cell populations to equal proportions of *P* and *M* cells (Fig 1B). Here, screening specifically for mutants that displayed biased cell-type ratios, differing by > 3 standard deviations from the mean, we isolated 105 candidates with skewed proportions (Fig 2B, S2 Fig, S2 Table). In several deletion strains, we detected co-expression of CFP and YFP in a cell. This phenotype was most likely caused by derepression of the mating-type information at *mat2-P* and *mat3-M* in these mutants [42]. In addition, 568 strains that could not be evaluated due to low fluorescence intensity or poor growth were examined by iodine staining, a stain for *S*. *pombe* spores that can be used as diagnostic for *mat1* switching (Fig 2C, S3 Table). Wild-type *h*^*90*^ colonies are stained darkly by iodine vapors because of their high spore content while mutants with altered mating or sporulation are stained less. Here, 124 deletion strains among the strains tested showed a staining different from wild-type. They were analyzed by quantitative multiplex PCR for *mat1* content alongside the 108 candidates that had passed the fluorescence microscopy screening.

Analyzing the content of *mat1* permitted us to pinpoint mutants for which biased cell-type expression or poor sporulation is likely to result from switching defects. A number of candidates failed to show a biased *mat1* content by multiplex PCR according to the chosen thresholds of P band intensity (Fig 2A and 2D). Indeed, many mutations might result in biased reporter gene expression or altered sporulation without affecting mating-type switching, for example genes located in the L region of the *mat* locus were eliminated from this screening. In addition, 35 deletion mutants that were diploid and/or heterothallic (*h*^*+N*^) were eliminated from the list of candidates (S4 Table). A bias in *mat1* content was detected for 32 mutants, in all cases towards *mat1-M*, suggesting an increased use of *mat3-M*. The identity of the deleted gene in these mutants was confirmed using published barcode sequences or gene specific primers. Surprisingly, 9 genes encoding ribosomal proteins are in the list, possibly as a consequence of protein synthesis defects. We did not pursue the investigation of these mutants, but focused instead on the remaining 23 mutants. These included nearly all known non-essential switch-related genes, 16 of which were identified in total. A few switch-related genes (*swi1*, *5* or *10*) were not deleted in the Bioneer library and were therefore not tested by the screen. A few other mutants might have escaped detection due to clonal variation, alternative switching phenotype with low frequency as for *msc1Δ* [49] or due to a switching bias close to the set thresholds, as for *mrc1Δ* [16]. Globally though the screening strategy was strongly validated by the identification of known switch-related factors alongside novel factors. The 7 newly identified factors include the F box protein Pof3, the CK2 family regulatory subunit Ckb1, the elongator complex subunit Elp6, the E3 ubiquitin protein ligase Brl2 and three subunits of the Set1/compass complex (Set1C), Swd1, Swd2 and Spf1 (Fig 2D, S2 Fig, Table 1). A protein interaction network analysis regrouping novel and previously known factors showed a high degree of connectivity (see below).

The fluorescence microscopy and multiplex PCR analyses for known and newly identified factors (S2 Fig) were confirmed by Southern blot analysis of *mat1* content with the *Dde*I restriction enzyme (S3 Fig).

### Classification of genes related to mating-type switching by Southern blot

**Fig 3:**
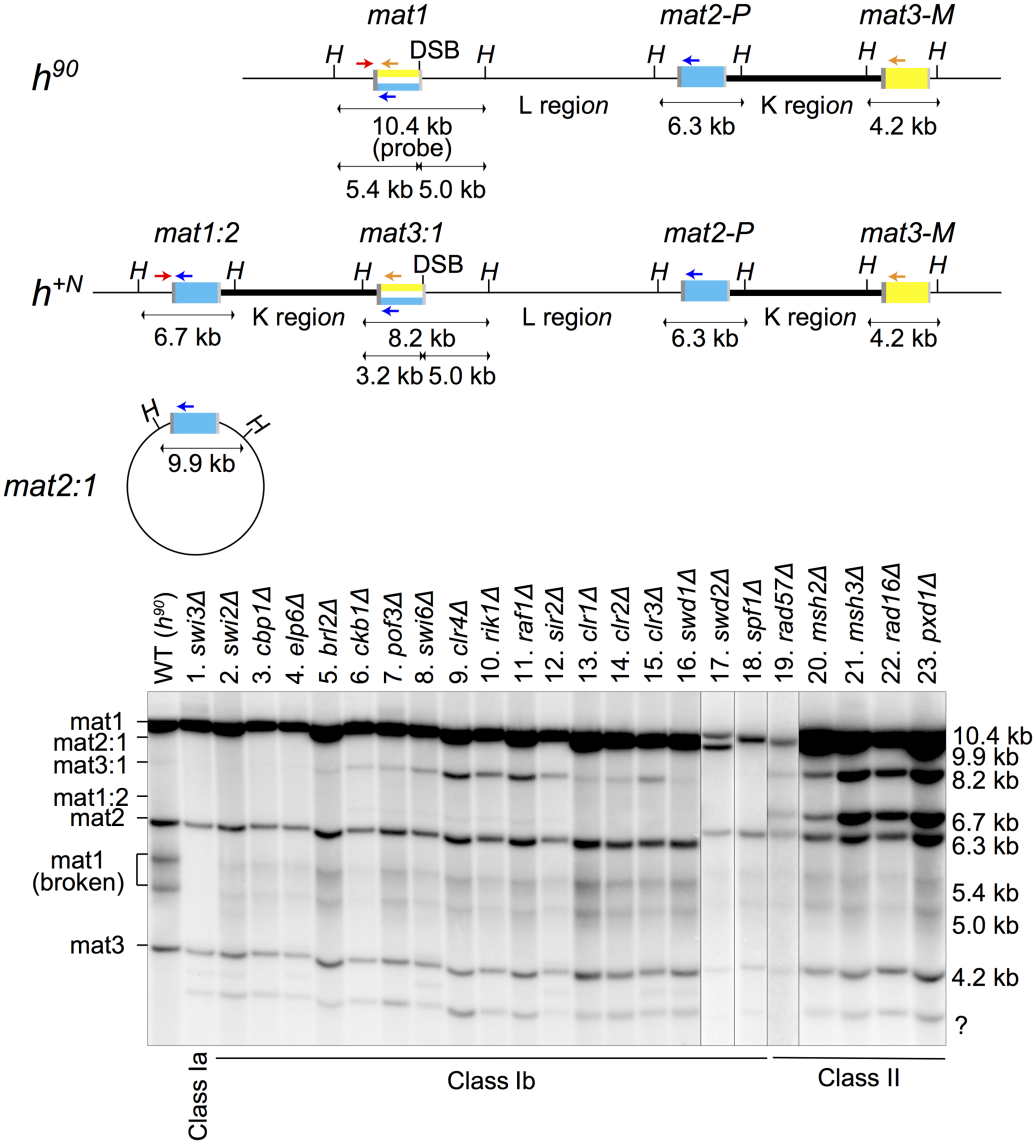
Classification of mutants obtained in the screen according to imprint formation and rearrangements. Southern blot analysis of *Hin*dIII-digested DNA. The probe was made from a 10.4 kb *mat1 Hin*dIII fragment. The positions of *Hin*dIII sites and primers used for PCR in S3 Fig (red arrow: *mat1*-specific; yellow: M-specific and blue: P-specific) are indicated above the blots. Strain PG4045 shows the hybridization pattern of a wild-type *h*^*90*^ strain. Mutants were classified according to imprint level and occurrence of specific rearrangements. The 5.4 kb and 5.0 kb bands are products of the DSB that occurs at the imprinting site during DNA preparation; they are absent in the Class Ia mutant, *swi3Δ*. *mat3*:*1* (8.2 kb) and *mat1*:*2* (6.7 kb) result from the *h*^*+N*^ rearrangement of the mating-type region. These fragments are present in Class II mutants. The 8.2 kb band in Class Ib mutants might originate from a *mat3*:*1* extrachromosomal circular element, or from a duplication of the mating-type region resulting from unequal sister chromatid exchange between *mat1* and *mat3*. *mat2*:*1* (9.9 kb) might result from a circular minichromosome in the Bioneer *swd2Δ* mutant. ‘?’ marks a band of unknown origin.

Southern blots can be used to detect the imprint at *mat1* and to determine whether rearrangements have occurred in the mating-type region. A DSB results from breakage at the fragile imprint site during DNA preparation [40]. As mentioned in the Introduction, the mating-type switching factors can be subdivided into three groups by Southern blot analysis of mutants, reflecting the molecular function of each factor. Class Ia is required for DSB formation at *mat1*, Class Ib is involved in donor selection for mating-type switching or other steps in the use of the break, and Class II is required for processing the gene conversion intermediates [40]. The 23 strains selected for analysis were assayed by Southern blot (Fig 3). The analysis confirmed previous conclusions in the case of known factors, for instance *swi3Δ* abolished DSB, and *rad57Δ*, *msh2Δ*, *msh3Δ*, *rad16Δ*, and *pxd1Δ* caused high frequencies of rearrangements of the *h*^*+N*^ type as expected for resolution-defective mutants [40]. The newly identified mating-type switching genes were assigned to Class Ib; *elp6Δ*, *swd1Δ*, *spf1Δ*, *brl2Δ*, *pof3Δ* and *ckb1Δ* strains were in that category together with *swi2Δ*, *cbp1Δ*, *swi6Δ*, *sir2Δ* and deletions of the Clr4 methyltransferase complex (CLRC), *clr4Δ*, *rik1Δ* and *raf1Δ*, or Snf/Hdac-containing repressor complex (SHREC), *clr1Δ*, *clr2Δ* and *clr3Δ*, subunits. As previously noticed for the *swi6Δ* mutant [51] a rearrangement producing an 8.2 kb *Hin*dIII fragment could be detected in several Class Ib mutants. The rearrangement could be a *mat3*:*1* circle or a duplication of the mating-type region creating a *mat3*:*1* cassette: *mat1-L-mat2-P-K-mat3*:*1-L-mat2-P-K-mat3-M*. The *mat3*:*1* cassette would not be amplified by the primers used to detect *mat1* content by PCR. The *swd2Δ* strain differed from the other mutants by showing a 9.9 kb *Hin*dIII fragment hybridizing to the *mat1* probe, however reconstruction of the strain produced a Class Ib mutant lacking this additional band and the reconstructed deletion allele was used in further analyses.

### Investigations of directionality defects in *h*^*09*^ cells and in cells with mutated recombination enhancers

**Fig 4:**
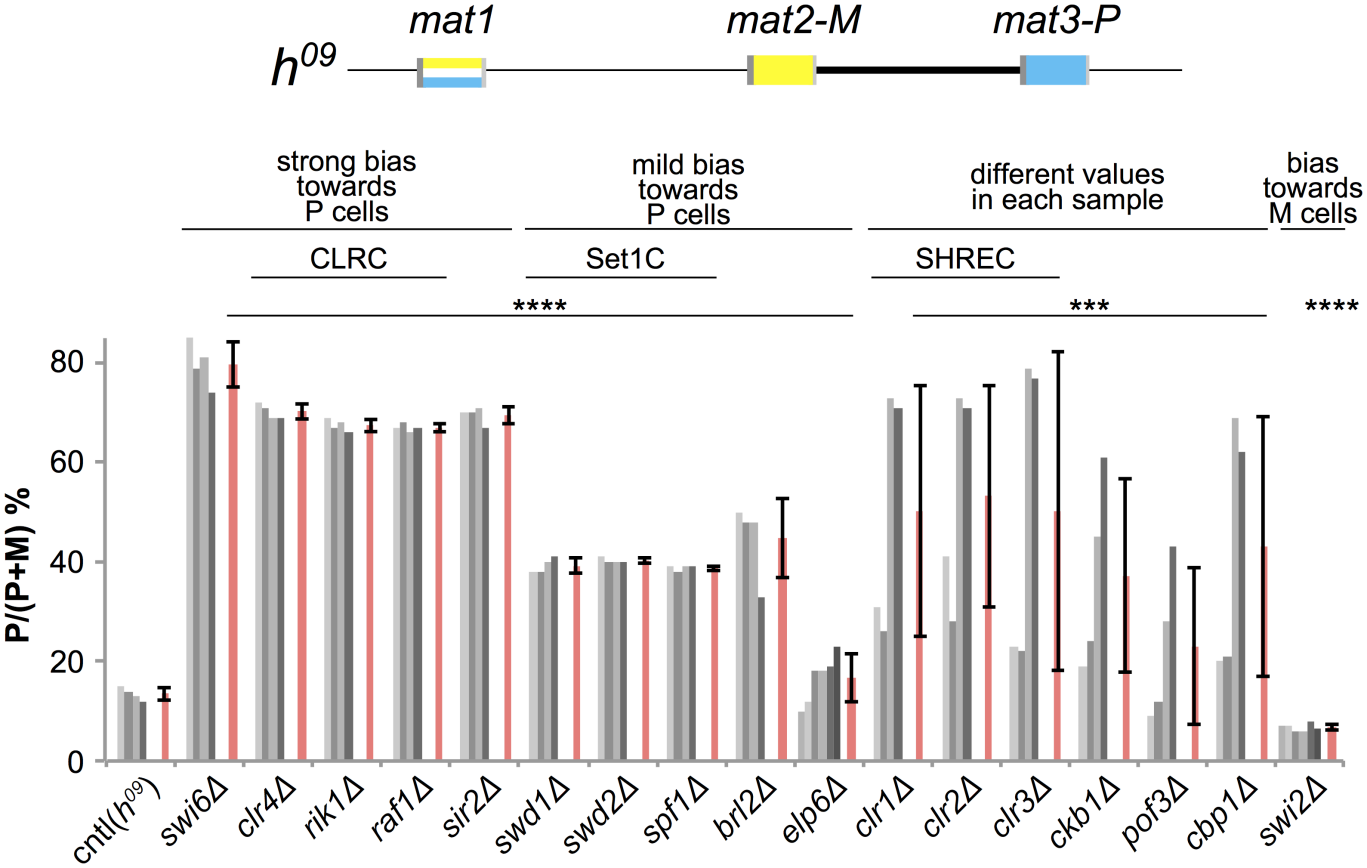
Determination of donor-choice preferences in mutants with the *h*^*09*^ mating-type region. The donor loci in *h*^*09*^ strains are swapped from *mat2-P mat3-M* to *mat2-M mat3-P*. Changes in *mat1* content caused by the Class Ib mutations identified here were estimated by multiplex PCR in the *h*^*09*^ background, from the band intensities shown in S4 Fig. Red bars represent means ± SD. Clr4, Raf1 and Rik1 are components of the H3K9 methyltransferase complex CLRC. Sir2 is a deacetylase. Swd1, Swd2 and Spf1 are components of H3K4 methyltransferase complex, Set1/Compass, Clr1, Clr2 and Clr3 are components of the deacetylase complex SHREC. One-way ANOVA was used to compare means of each sample to cntl, ***p < 0.001; ****p > 0.005.

In the *h*^*09*^ mating-type region, the contents of the silent cassettes are swapped to *mat2-M mat3-P* (Fig 4, S4 Fig). This arrangement results in inefficient heterologous switching and in a *mat1* content biased towards *mat1-M* [3]. How mutations affect this bias provides insights into the directionality of mating-type switching. For example, deletion of *swi6* biases the *mat1* content towards *mat1-P* in *h*^*09*^ cells, and towards *mat1-M* in *h*^*90*^ cells, consistent in both cases with preferred selection of *mat3* as a donor [3]. This likely reflects a preferential use of the *SRE3* recombination enhancer in *swi6*Δ cells [23]. We created *h*^*09*^ strains to test whether the newly identified Class Ib factors contribute to mating-type switching in the same or similar way as Swi6. Each deletion mutant was crossed with the *h*^*09*^ strain PG4048. After the selection of recombinants, we analyzed four independent colonies of each *h*^*09*^ deletion strain by multiplex PCR for *mat1* content. As a control, colonies originating from spores of self-mating PG4048 cells were analyzed. As expected, PG4048 contained a greater proportion of M cells than P cells (a mean of 86% M cells; Fig 4). All mutations tested affected this ratio. The effects varied. During these analyses, we created a fresh deletion of *swd2* to eliminate the rearrangement detected in the Bioneer mutant (9.9 kb band in Fig 3), as mentioned above, and confirmed that the observed directionality defects were not a result of the rearrangement.

A first group of factors comprised Swi6, CLRC subunits (Clr4, Rik1 and Raf1) and the histone deacetylase Sir2 (Fig 4). Mutations affecting CLRC or Sir2 produced nearly identical values (around 70% P cells), similar to the loss of Swi6 (77% P cells). These mutants were also very similar to each other in the *h*^*90*^ background (around 20% P cells; Fig 2D). In the mating-type switching process, CLRC and its catalytic Clr4 subunit are believed to work by catalyzing the methylation of H3K9 in the *mat2*-*mat3* heterochromatic domain and creating binding sites for Swi6 [24, 52–55]. The role of Sir2 in the process might be to remove acetyl groups from H3K9 [46], thus facilitating heterochromatin formation [46, 56–58]. Indeed, the methylation of H3K9 and Swi6 association are reduced several fold in the mating-type region of *sir2Δ* cells [46]. This is consistent with Sir2 acting upstream of CLRC and Swi6 through H3K9 deacetylation, without excluding that other actions of Sir2, e.g H3K4 deacetylation [59], might also be relevant to directionality.

**Fig 5:**
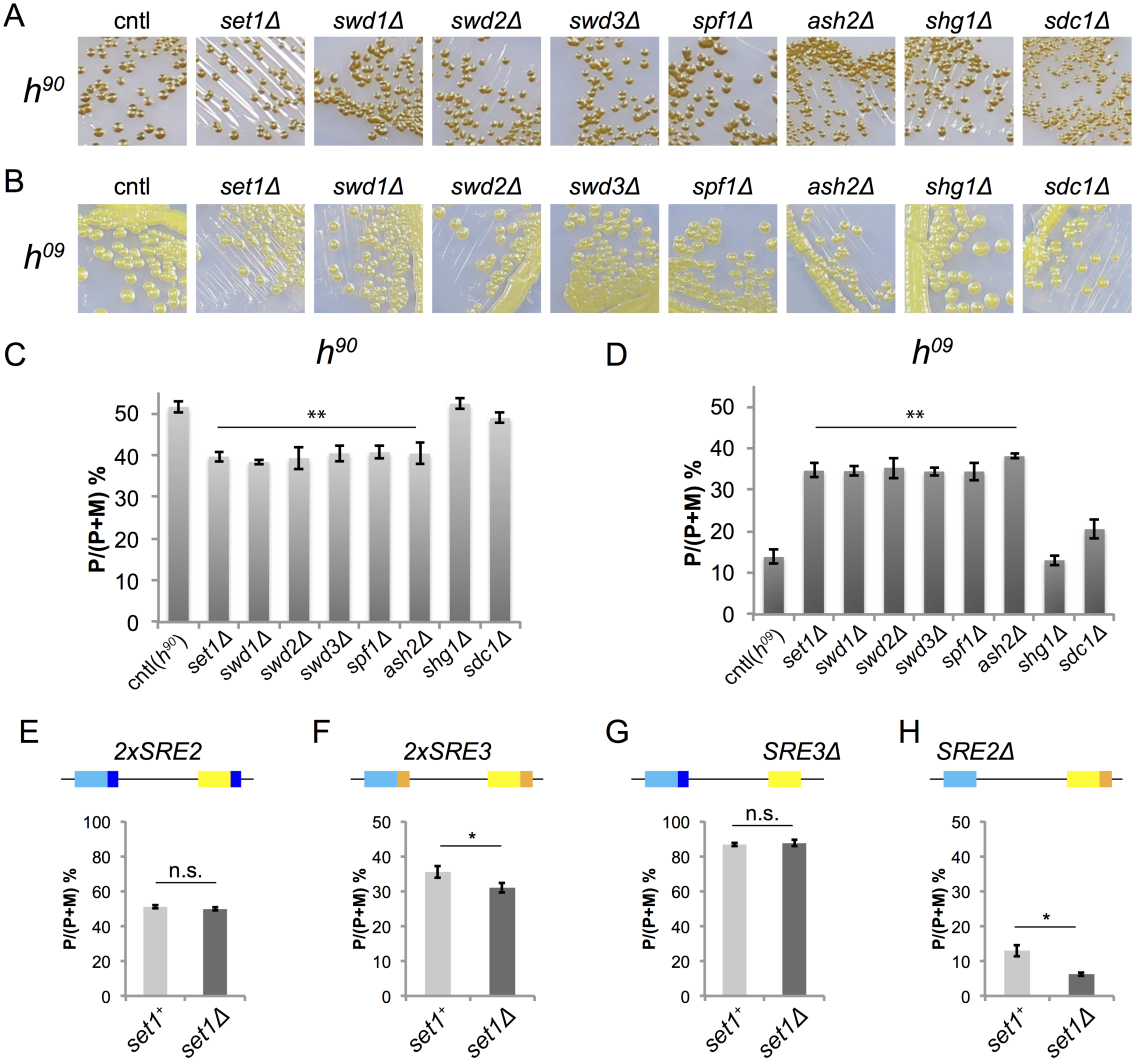
Analysis of mating-type switching directionality of Set1C mutants. (A, B) Spore formation of mutants in each Set1C subunit was assayed by exposure of colonies to iodine vapor. *h*^*90*^ strains are shown in (A) and *h*^*09*^ stains are shown in (B). (C, D, E) Quantification of *mat1* content estimated by multiplex PCR. The relative P band intensity in each lane (P/(P+M)) was calculated from the data shown in S5 Fig for the *h*^*90*^ strains shown in (C); from S5 Fig for the *h*^*09*^ strains shown in (D) and from S5 Fig for the strains with mutated *SRE* elements shown in (E-H). (E) 2×*SRE2* strains (*mat2-P-SRE2 mat3-M-SRE2*) were derived from the *set1*^+^ strain TP126; (F) 2×*SRE3* strains (*mat2-P-SRE3 mat3-M-SRE3*) from TP303; (G) *SRE3Δ* from TP75; and (H) *SRE2Δ* from TP8. Four *set1*Δ isolates are shown in each case. (C-H) Data are represented as mean ± SD. Two-tailed paired Student’s t test was used to compare the mean of each sample to cntl, *p < 0.01; **p < 0.005; n.s., not significant.

The second group of mutants affecting mating-type switching in *h*^*09*^ cells were deletions of the genes for the ubiquitin E3 ligase Brl2, the Set1C subunits Swd1, Swd2 and Spf1, and the Elongator subunit Elp6 (Fig 4). These mutants resulted in a consistent increase in *mat1-P* content in *h*^*09*^ cells, from ~20% in wild-type background to ~35% in each of the three mutants. While not as pronounced as for Swi6 or CLRC mutants, the increase occurred to the same degree in all four isolates examined in each case. These mutants were unexpected because it has been reported that *set1Δ* has no effect on mating-type switching [60]. We examined each component of Set1C (Set1, Swd1, Swd2, Swd3, Spf1, Ash2, Shg1 and Sdc1 [61]) by iodine staining and multiplex PCR of mutants (Fig 5A–5D). Consistent with the previous report [60], deletion of *set1* or other subunit genes showed little effect on iodine staining of either *h*^*90*^ or *h*^*09*^ colonies (Fig 5A and 5B). However, monitoring *mat1* content clearly showed that individual gene deletions biased switching toward *mat3-P* in *h*^*09*^ cells and resulted in a correlated increased use of *mat3-M* in *h*^*90*^ cells for six Set1C subunits (Fig 5C and 5D). This trend is similar to mutations in the H3K9 methylation pathway, although not to the same amplitude (Fig 4). Interestingly, the iodine staining level of *set1Δ* colonies differed between *h*^*90*^ and *h*^*09*^ in spite of similar cell-type ratios (39% P cells in *h*^*90*^ and 36% in *h*^*09*^). Due to a different switching pattern, P and M cells might be less evenly mixed in *h*^*09*^ colonies compared with *h*^*90*^. This would lead to less efficient mating and spore formation in *h*^*09*^ even though the cell-type bias is only a little more pronounced than in *h*^*90*^. To investigate the functionality of each *SRE* element in *set1Δ* cells, we analyzed directionality in *SRE* element mutants (Fig 5E–5H). Deletion of *set1*^+^ did not significantly affect *mat1* content in 2×*SRE2* cells (where the *SRE3* element is replaced with *SRE2*) (Fig 5E) or in *SRE3Δ* cells (Fig 5G). However, in populations of *2*×*SRE3* cells (where the *SRE2* element is replaced with *SRE3*) deletion of *set1* caused a small increase in M cells (31% P cells in *2*×*SRE3 set1Δ* compared with 36% P cells in *2*×*SRE3 set1*^+^) (Fig 5F). In the case of *SRE2Δ*, donor choice was more strongly biased towards *mat3-M* in *set1Δ* cells (6% P cells) than in *set1*^+^ cells (13% P cells) (Fig 5H). This suggests that Set1C normally inhibits the choice of *mat3-M*-*SRE3* in M cells. It has similarly been observed that deletion of *swi6* causes virtually no change in donor choice in the 2×*SRE2* and *SRE3Δ* backgrounds, where *SRE2* keeps being used, but decreases use of *SRE3* in *SRE2Δ* cells (5% P cells in *SRE2Δ swi6Δ* compared with 16% P cells in *SRE2Δ swi6*^+^) [23]. Loss of the Brl2 ubiquitin ligase resulted in phenotypes similar to mutations compromising Set1C (Figs 4 and 5). Thus, like Swi6 and CLRC, Set1C and Brl2 appear to favor use of the *SRE2* recombination enhancer over *SRE3* when both enhancers are present, perhaps by inhibiting the use of *SRE3*, to result in balanced switching.

A third group of mutants displayed a variegated phenotype (*clr1Δ*, *clr2Δ*, *clr3Δ*, *ckb1Δ*, *pof3Δ* and *cbp1Δ*; Fig 4), with the proportion of P cells varying between independent cultures. In some isolates, the proportion of P cells was nearly wild-type whereas in others it was similar to the *swi6Δ* mutant. These phenotypes were not caused by rearrangements in the mating-type region (S4 Fig). Clr1, 2 and 3 are subunits of SHREC, the Snf2-histone deacetylase repressor complex [62]. Clr3 participates in the recruitment of Clr4 to the mating-type region, but in its absence a Clr3-independent, RNAi-dependent pathway accomplishes this function to some extent [63]. Clr3 localizes to three regions in the mating-type region, which are close to *mat2-P* (*RE*II), *cenH* and *mat3-M* (*RE*III), respectively [62, 64]. In the *cenH* region, the heterochromatin platform is likely established by RNAi-mechanisms; on the other hand, at the *RE*III site, it is established by an RNAi-independent mechanism [63, 65, 66]. The clonal variations observed in *clr1*, *clr2* and *clr3 h*^*09*^ mutants might reflect these distinct pathways of heterochromatin establishment similar to position effect variegation [67]. Heterochromatin would be partially formed and inherited in some clonal populations of the mutant strains but not in others, leading to populations differentially proficient for switching. It is also known that the CK2-dependent phosphorylation of Swi6 mediates Clr3 recruitment to centromeric regions [68], possibly accounting for the Ckb1 defects observed here.

### Swi6-dependent and independent pathways of mating-type switching

**Fig 6:**
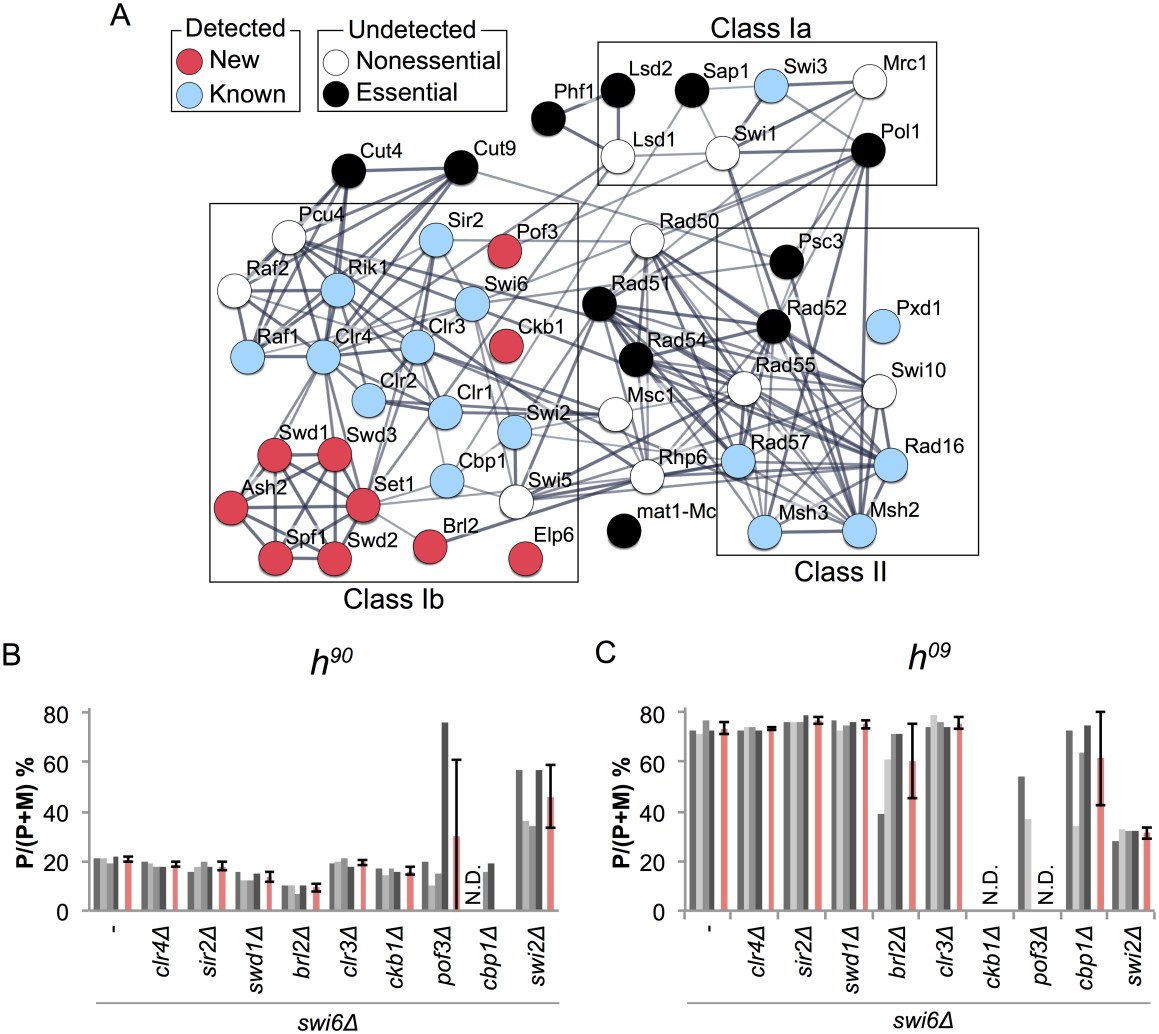
Protein interaction network and genetic interactions. (A) An interaction network of newly identified and previously known mating-type switching factors (Table 1) was obtained from the STRING database (v10.0). The proteins are represented by nodes. Red and blue nodes show factors detected in this screen. White and black nodes show known mating-type switching factors that were not detected in this screen, either because the gene deletions were not in the library, or due to the set thresholds or human error. The line thickness represents the strength of the association (confidence > 0.6). It has been suggested that the presence of the DSB at *mat1* is lethal in deletion mutants of *rad51*, *rad52 and rad54* [36, 38]). (B, C) Genetic interactions between *swi6* and the identified Class Ib genes. *mat1* content was quantified for the indicated double mutants with the *h*^*90*^ (B) or *h*^*09*^ (C) mating-type region. The relative P band intensities (P/(P+M)) were calculated from gels shown in S6 Fig. Red bars represent means ± SD.

To further address the mechanisms of mating-type switching, we analyzed the protein-protein interaction network linking newly identified and previously known mating-type switching factors in the STRING database (Table 1) [69]. The obtained interaction network correlates strongly with categories established by Southern blotting and phenotypic classification (Fig 6A). Among the newly identified factors, six subunits of Set1C and Brl2 are connected to Class Ib factors. While Pof3, Ckb1 and Elp6 show no direct interaction with Class Ib factors in the STRING analysis (Fig 6A), several studies have reported that Pof3 plays a role in heterochromatin silencing [70–72] and Ckb1 phosphorylates Swi6 [68]. Elp6 is an orthologue of a part of the six-subunit Elongator complex (Elp1-6) in *S*. *cerevisiae* [73, 74]. Elp3 also passed the initial screening (S2 Table), however the other subunits of Elongator complex did not. The main cellular function of Elongator is thought to be in tRNA modification, but Elongator has also been proposed to acetylate histones [75–77].

We tested the hypothesis that some factors might act through Swi6 by performing an epistasis analysis. Double mutants combining *swi6Δ* with each candidate gene deletion were constructed and four independent colonies were analyzed by multiplex PCR for each of them (Fig 6B and 6C).

**Fig 7:**
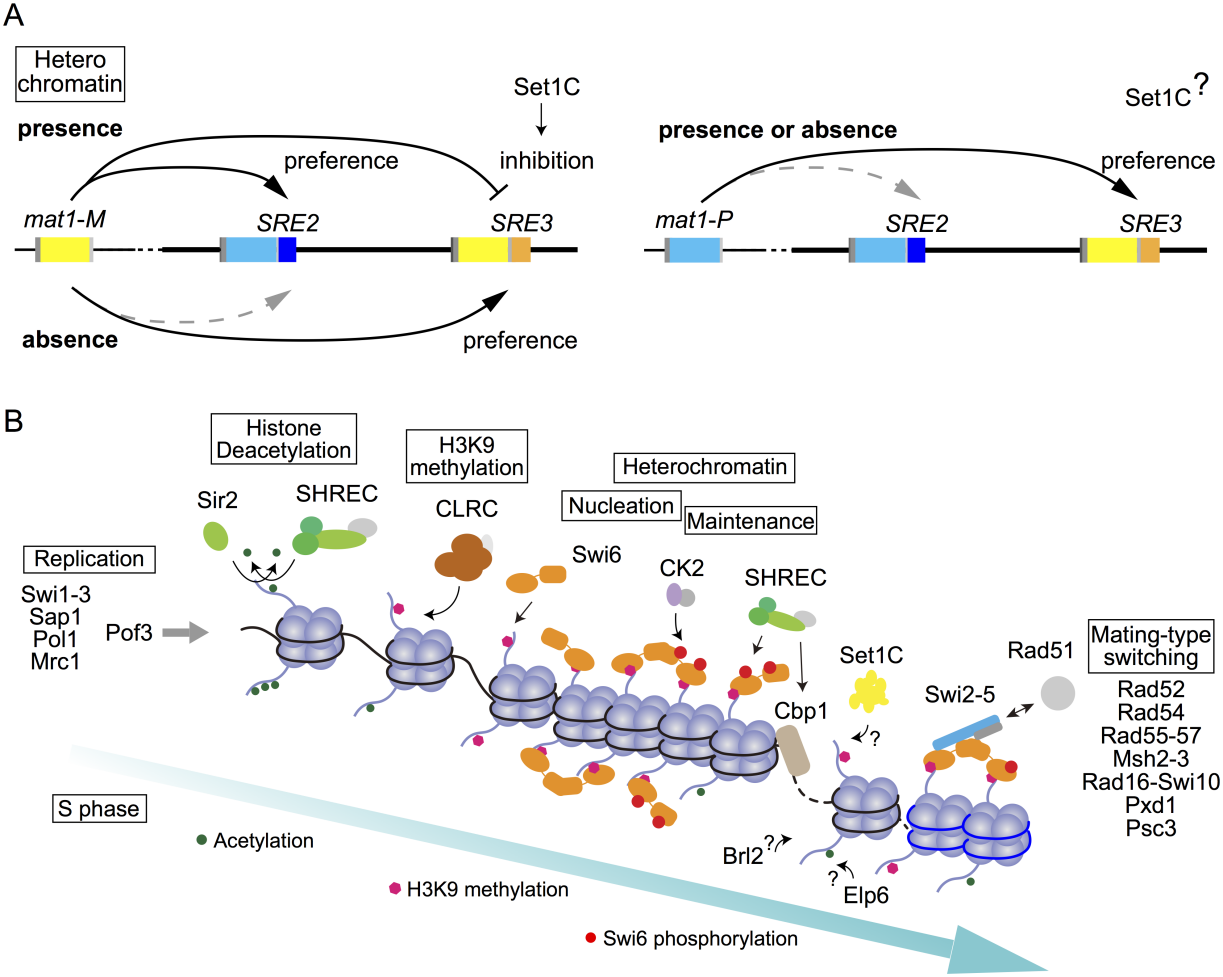
Mating-type switching model. (A) Donor selection controlled by heterochromatin. In the presence of heterochromatin, *mat2-P* is preferred over *mat3-M* in M cells due to an increased accessibility of *SRE2* to switch-promoting binding factors and to a reduced accessibility of *SRE3*. In the absence of heterochromatin, *SRE3* can stimulate recombination by Swi6-independent binding of Swi2. In P cells, the inhibition to select *SRE3* is released. (B) Model of mating-type switching regulated by heterochromatin formation. The histone deacetylases Sir2 and SHREC remove histone acetylation (green). CLRC methylates H3K9 (pink). Swi6 binds to methylated H3K9 and nucleates heterochromatin. CK2 phosphorylates Swi6 (red). Cbp1 binds at specific regions in the *mat* locus. Clr3 (SHREC) is recruited to phosphorylated Swi6 and Cbp1 binding site. Set1C might affect heterochromatin at *SRE3*. The Swi2-5 complex binds to Swi6 and Rad51, which regulates mating-type switching directionality. Pof3, Elp6 and Blr2 might also affect switching by histone modification.

The mating-type switching phenotypes of the *swi6Δ clr4Δ* and *swi6Δ sir2Δ* double mutants were quite similar to the single deletions of *swi6*, *clr4* or *sir2* (Figs 2D, 4, 6B and 6C). This is consistent with CLRC and Sir2 being required for heterochromatin establishment and Swi6 recruitment at the *mat* locus (Fig 7).

Double mutants combining *swi6Δ* with *swd1Δ*, lacking a Set1C subunit, or *clr3Δ*, lacking a SHREC subunit, also displayed a phenotype quite similar to the *swi6Δ* single mutant (Fig 6B and 6C). This indicates that Set1C and SHREC work in the Swi6 pathway. A previous study has shown that *set1Δ* does not affect Swi6 localization or silencing of a *ura4*^+^ marker gene at *cenH* in the mating-type region [60] but other assays have found that Set1C subunits participate in the repression of heterochromatic loci including the mating-type region [78]. This latter effect might be related to the occurrence of switching defects in Set1C mutants. Set1C may control Swi6 localization in a site-specific manner such as at *SRE3* (Fig 5).

Donor preference in *swi6Δ ckb1Δ* cells indicated that *swi6Δ* is also epistatic to *ckb1Δ* even though phenotypes could only be assigned in the *h*^*90*^ background due to apparently high rates of rearrangement in the *h*^*09*^ background. Nevertheless, the data suggest that Swi6 phosphorylation by Ckb1 [68] is important for switching directionality controlled by Swi6.

More complex epistatic relationships were observed for the remaining mutants, *brl2Δ*, *pof3Δ*, *cbp1Δ*, *elp6Δ*, and *swi2Δ*, when these mutations were combined with *swi6Δ* (Fig 6B and 6C). The predominant mating-type had a tendency to vary between isolates, particularly with the *h*^*09*^ mating-type region, and rearrangements occurred. Double mutants combining *brl2Δ* and *swi6Δ* showed a *mat1-M* bias apparently even more pronounced than for the *swi6Δ* single mutant in *h*^*90*^, while in *h*^*09*^ two *swi6Δ brl2Δ* isolates were similar to *swi6Δ* and two had more balanced *mat1* contents. Populations of *h*^*90*^ *swi6Δ pof3Δ* cells showed biases similar to the *h*^*90*^ *swi6Δ* single mutant for three isolates, whereas the fourth isolate was biased towards P cells rather than M cells. Populations of *h*^*09*^ *swi6Δ pof3Δ* cells had varied ratios of P and M cells, and two isolates were rearranged. The switching bias for *swi6Δ cbp1Δ* was similar to *swi6Δ* with the *h*^*90*^ mating-type region (Fig 6B), but two strains in four differed from *swi6Δ* with the *h*^*09*^ mating-type region (Fig 6C). This phenotype may be caused by Swi2 expression level, which is controlled by Cbp1 [26, 31]. Rearrangements in *h*^*90*^ *swi6Δ elp6Δ* and *h*^*09*^ *swi6Δ elp6Δ* mutants precluded analysis. These observations suggest that both Swi6-dependent and -independent pathways control switching directionality by Brl2, Pof3 and Cbp1. Finally, *swi2Δ swi6Δ* double mutants differed from *swi6Δ* in both *h*^*90*^ and *h*^*09*^. It has been reported that Swi2 can localize to *SRE3* in the absence of Swi6 [22, 23, 27]. The more balanced cell populations in *swi6Δ swi2Δ* mutants are probably caused by loss of Swi6-independent function of Swi2.

In summary, our observations suggest that the factors, Clr4, Sir2, Swd1 and Clr3 probably work in the same pathway as Swi6, but Brl2, Pof3, Cbp1 and Swi2 have an effect on donor selection through Swi6-dependent and -independent mechanisms. Frequent DNA rearrangements in Class Ib mutants (Fig 3) and in double mutants with *swi6*Δ indicate that histone modifications not only direct donor choice, but also facilitate resolution steps or prevent unequal sister chromatid exchanges between cassettes.

### Mating-type switching model by regulation of Swi6 association with the mating-type region

We propose a model summarizing how each factor identified in this study might participate in the donor selection mechanism (Fig 7). In this model, the heterochromatin structure in M cells favors the cassette adjacent to *SRE2* as a donor while structural changes in mutants and in P cells favor *SRE3* [23]. In addition, chromatin structure prevents selection of the cassette adjacent to *SRE3* in M cell (Fig 7A). It has been reported that *SRE2* can facilitate donor choice efficiently not only in M cells but also in P cells whereas S*RE3* is more active in P cells than in M cells [23]. These data indicate that the inhibition of donor choice does not affect *SRE2*.

Mating-type switching is initiated by a site-specific imprint during replication. In the replisome, Swi1-Swi3, Pol1 and Mrc1 are required for the imprint [16, 40]. One of the novel switch factors identified here is the ubiquitin ligase component Pof3. Pof3 interacts with the replisome, in an Mrc1- and Mcl1-dependent manner [71, 79]. However, rather than participating in imprint formation, we found that Pof3 affects donor selection. Pof3 is also required for heterochromatic silencing near *mat3* [70]. We speculate that both effects are brought about by the Pof3-mediated degradation of replisome components [80] or of Ams2, a cell cycle-regulated transcription factor for histone genes [81] that also mediates long range chromosomal interactions [82] and interacts with Raf1, a component of CLRC [83]. Thus, Pof3 would couple the deposition of new histones and their modification by CLRC. During S phase, partly as a result of new histones deposition onto replicated DNA, Swi6 and H3K9me2 levels decrease at silenced loci [84, 85]. A wave of H3K9 acetylation, observed in other organisms in front of the replication fork [86], might further weaken heterochromatin. The current search expands on previous work to show that enzymatic complexes required for the restoration of heterochromatin, both NAD^+^-dependent and -independent HDACs and CLRC, are necessary for donor selection. Remarkably, lack of Sir2 or of a CLRC subunit phenocopied the *swi6* deletion in both *h*^*90*^ and *h*^*09*^ cells (Figs 2D and 4) while the loss of SHREC components resulted in variegated phenotypes. We take these differences as reflecting the different substrate specificities and recruitment mechanisms of the two HDACs to heterochromatic regions [57, 87]. In both cases, our epistasis analysis with the Swi6 mutant points to defects in Swi6 recruitment. Our search also identified Cbp1 and CK2, both of which are thought to recruit Clr3 to heterochromatin regions [64, 68]. Cbp1 also controls expression of the *swi2* gene and the cell-type specific protein isoform it produces, together with the M-specific protein Mc [26, 31].

A novel and intriguing outcome of our study is that multiple subunits of Set1C and the E3 ubiquitin ligase Brl2 are required for accurate donor selection. Set1C catalyzes the methylation of H3K4. A role in heterochromatin appears paradoxical, given that methylated H3K4 is strongly associated with expressed genes. One possibility is that Set1C regulates the expression of Swi2, central effector of switching directionality, or of other switching factors. RNA profiling analysis has revealed that switching genes are expressed to similar levels in wild type and individual Set1C mutants [87], however more subtle effects such as shifts in transcription initiation have not been ruled out. In addition, *S*. *pombe* Set1C appears directly required for silencing at various locations [78]. In the mating-type region, all subunits except for Shg1 are required for silencing of the *cenH* repeat. Evidence has also emerged for roles in meiotic recombination [88, 89] even though the effects of H3K4 methylation on recombination have been hard to unravel due to the prevalence of that modification genome-wide [90]. Here, we favor a simple model where Set1C affects donor choice directly. This could be through local, possibly temporally restricted methylation of H3K4 at *SRE3* that would either act as such or by preventing H3K4 acetylation. Brl2 is part of HULC that catalyzes the ubiquitylation of histone 2B (H2Bub) [61, 91, 92]. H2Bub stabilizes the interaction of Set1 with chromatin *in vitro* [93]. The single *brl2Δ* deletion affected *mat1* content similar to the deletion of Set1C components (Figs 2, 4 and 5). These connections and phenotypic similarities indicated that Set1C and HULC might co-operate to choose a correct donor. (Figs 4 and 6B). However, the phenotypes of two in four independent colonies of *h*^*09*^ *swi6Δ brl2Δ* strain differed from *h*^*09*^ *swi6Δ swd1Δ* (Fig 6C). Brl2 is also known to interact with Nse5, which is a part of the structural maintenance of chromosome 5/6 (Smc5-6) holocomplex [94, 95]. It may have multiple functions in mating-type switching.

Following the cell-type specific deposition of Swi6, Swi2-Swi5 localizes to *SRE2* by interaction with Swi6 in M cells [22, 23, 27]. Together with the inhibited use of *SRE3*, this regulated recruitment of Swi2-Swi5 effectively directs the Rad51 strand-exchange protein to initiate homologous recombination at the proper cassette, providing an increasingly well understood model for the effects of chromatin structure on recombination.

## Materials and methods

### Yeast strains, strain construction, and strain manipulations

*S*. *pombe* strains were generated and propagated according to standard protocols [96]. They were manipulated with a Singer RoToR plate-handling robot (Singer Instruments) for high throughput screens. To test for mating-type switching defects by fluorescence analysis, a query strain (PG4045: *h*^*90*^ *(Blp1)*::*LEU2 leu1*::*ura4*^+^*-[mfm3p-YFP]-[map2p-CFP] ura4-D18 ade6-M216* was mated to the Bioneer gene deletion library (*h*^+^ *leu1-32 ura4-D18 ade6-210 or 216*, *ORFΔ*::*kanMX4*). Mating was performed on SPA plates supplemented with 200 mg/l leucine, 100 mg/l uracil, and 100 mg/l adenine. Cells were allowed to mate and sporulate at 30°C for two days. The mating plates were then moved to 42°C for three days to eliminate vegetative cells. Following heat treatment, spores were transferred onto YES plates with 100 mg/l G418 and allowed to germinate and divide for three days at 30°C. To select for *h*^*90*^ progeny, cells were then transferred from the YES plates to MSA plates with 100 mg/l G418 and 100 mg/l adenine and grown for a further three days at 30°C. This scheme selects for *(Blp1)*::*LEU2*, tightly linked to the *h*^*90*^ region, for *ura4*^+^, tightly linked to the fluorescent reporters, and for *kanMX4* that marks each ORF deletion. Single colonies were isolated from bulk recombinants by streaking cells onto MSA plates containing G418 and adenine, incubated for three days at 30°C. The *sdc1* deletion strain (*h*^+^ *sdc1*::*Kan*^*r*^ *ade6-M21*? *leu1-32 ura4-D18*) was obtained from the National BioResource Project (NBRP ID: FY23769, strain name: P1-1G).

### Fluorescence microscopy

Single colonies of recombinants obtained as described above were inoculated into 50 μL MSA medium supplemented with 100 mg/l adenine and grown for two days at 30°C in 96 well plates. Cells were then diluted 24 times into MSA medium with adenine and grown for ~20 hr at 30°C. Sixty μl cell suspensions diluted 15 times with MSA medium with adenine were transferred to 384 well microplates with clear bottom (CellCarrier-384 ultra, Perkin Elmer). The fluorescence of cells was measured using an Opera high-content screening microscope (Perkin Elmer). The following settings were used [Filter sets: Camera 1: 475/50 for CFP signals, Camera 2: 540/75 for YFP signals, Camera 3: 690/50 for bright field, Light source: 405/488/635]. Twelve images were taken for each well, for a total of ~200 cells per strain. The proportion of P cells was then calculated using the Acapella software program (PerkinElmer). Selected strains were imaged again using a Delta Vision Elite microscope (GE Healthcare).

### Iodine staining

Cells were plated onto MSA medium supplemented with 100 mg/l adenine, allowed to form single colonies, and exposed to iodine vapors.

### Multiplex PCR

*S*. *pombe* cells were propagated in 2 mL liquid YES cultures at 30°C to saturation. Genomic DNA was prepared from wild-type and mutant cells as described [97]. The genomic DNA concentration was measured using QuantiFluor One dsDNA Dye System (Promega) and 4 μL genomic DNA (1.25–5.0 ng/μL) in TE was added to 16 μL PCR reaction reagent (total 20 μL) to perform multiplex PCR to determine the genetic content of the *mat1* or *mat3* locus. The primers used were FAM-MT1 (5’-AAATAGTGGGTTAGCCGTGAAAGG-3’) at 400 nM, MP1 (5’-ATCTATCAGGAGATTGGGCAGGTG-3’) at 200 nM and MM1(5’-GGGAACCCGCTGATAATTCTTGG-3’) at 200 nM (S3 Fig). The 5’ end of FAM-MT1 and FAM-MT3 were modified with 6-carboxyfluorescein (FAM). To reduce non-specific PCR products, 400 nM heat-stable RecA protein from a thermophilic bacterium, *Thermus thermophiles*, and 400 μM ATP were included in the PCR reaction buffer (10 mM Tris-HCl pH 8.3, 50 mM KCl, 2.5 mM MgCl_2_) [98]. The following amplification program was used: 2 min at 94°C—27 x [30 s at 94°C—30 s at 55°C—1 min at 72°C]—5 min at 72°C. PCR fragments corresponding to *mat1-P* and *mat1-M* alleles were resolved on 5% polyacrylamide gels. Fluorescence was detected and quantified using Typhoon FLA9500 (GE Healthcare) and ImageQuant (GE Healthcare).

### Barcode sequencing

Each gene knockout in the Bioneer collection contains ‘up-tag’ and ‘down-tag’ sequences that provide a unique barcode for each knockout. To confirm the identity of the mutants identified in our screen, we amplified the KanMX4 region using the U1 primer (5’-CGCTCCCGCCTTACTTCGCA-3’) and D1 primer (5’- TTGCGTTGCGTAGGGGGGAT). The PCR products were then sequenced using cpn1 (5'-CGTCTGTGAGGGGAGCGTTT-3') to read the up-tag and cpc300 (5'-AGACCGATACCAGGATCTTGCC-3') to read the down-tag. The results were compared with the barcode list.

### Southern blots

*S*. *pombe* cells were propagated in 16 mL liquid cultures (YES) at 30°C to pre-saturation and genomic DNA for Southern blots was prepared as described above. Genomic DNA was digested with *Hin*dIII to classify the mating-type switching defective genes according to DSB formation or presence of rearrangements in the mating-type region (Fig 3), or with *Dde*I to assay *mat1* content (S3 Fig). The digested samples were electrophoresed in 0.7% agarose gels. The probe to analyze *mat1* content was a PCR product made with GTO-1369 (5’- GAGCCTACTGTTAATATAATAACATTATG-3’) and GTO-1370 (5’-CCTTCAACTACTCTCTCTTCTTTTCCTACCC-3’), corresponding to the centromere-proximal *Dde*I-*Nsi*I fragment [23]. The probes to classify the mutants were 10.4 kb *Hin*dIII fragments containing *mat1-P* or *mat1-M*.

## Supporting information

S1 Fig Outline of screen. The Bioneer collection of *S*. *pombe* haploid deletion strains (V5) was mated with PG4045 on SPA plates in an arrayed format using a high-throughput robot (Singer Inst.). Spores were selected, germinated, and G418-resistant progeny that were also prototrophic for leucine (selecting for *h*^*90*^ mating-type region) and uracil (selecting for cell-type specific fluorescent reporters) were obtained, colony purified, re-arrayed, and examined by a combination of fluorescence microscopy with a high throughput Opera microscope (all strains), iodine staining of colonies (subset of strains with unclear fluorescence output), and multiplex PCR (subset of strains with potential mating-type switching defects obtained in the first screens).
(TIF)

S2 Fig Analysis of mating-type switching related genes by fluorescence microscopy and iodine staining assays. (A) Merged channels for wild-type *h*^*90*^ strain (cntl) and two mutants. The 15 mutants shown in (B, C) were identified by fluorescence microscopy or iodine staining analysis in the screen outlined in S1 Fig. (B) Repeat of cell-type quantification using a DeltaVision Elite microscope (GE Healthcare). (C) Iodine staining of strains with the indicated gene deletions.
(TIF)

S3 Fig Effects of culture conditions and detection method (multiplex PCR *versus* Southern blot) on *mat1* content measurements. (A, B) Representation of the *mat1* region showing (A) priming sites used for multiplex PCR and (B) the restriction sites and probe (*Dde*I-*Nsi*I fragment) used for the Southern blots in (E). (C) Location of primers and probe complementarity on *h*^*90*^, *h*^*+N*^, duplicated mating-type region (*mat1-L-mat2-P-K-mat3*:*1-L-mat2-P-K-mat3-M*), and *mat2*:*1* or *mat3*:*1* circles. (D) 30 μL of saturated YES pre-cultures (2 ml each) were used to inoculate 20 mL YES cultures that were then propagated until late exponential phase. DNA was extracted from both the 2 ml pre-cultures and the 20 ml cultures and analyzed by multiplex PCR. (E) DNA preps from 20 mL YES cultures were analyzed by Southern blot taking advantage of the size difference between a *mat1-P* and *mat1-M* fragment in *Dde*I digests. Measurements by multiplex PCR and Southern blots are compared.
(TIF)

S4 Fig Effect of Class Ib mutants identified in the screen on mating-type switching in the *h*^*09*^ strain. (A) Multiplex PCR analysis of the mutants shown in Fig 4. The content of *mat1* was estimated by quantification of P- and M-specific band intensities. (B) Southern blot analysis using *Hin*dIII digests of genomic DNA and a 10.4 kb *mat1 Hin*dIII fragment as probe.
(TIF)

S5 Fig Multiplex PCR analysis of mating-type switching directionality in Set1C subunit mutants and *SRE* element mutants. (A, B) Multiplex PCR analysis of the mutants shown in Fig 5C and 5D and quantification of *mat1* content estimated from P- and M-specific band intensities. The relative P band intensity in each lane (P/(P+M)) was calculated from (A) *h*^*90*^ and (B) *h*^*09*^. (C-F) Epistasis analysis by multiplex PCR analysis for the Set1 and *SRE* elements mutants shown in Fig 5E–5H. The relative P band intensity (P/(P+M)) was calculated for each lane. (C) 2×*SRE2* (*mat2-P-SRE2 mat3-M-SRE2*) strains were derived from strain TP126; (D) 2×*SRE3* (*mat2-P-SRE3 mat3-M-SRE3*) strains were derived from strain TP303; (E) *SRE3Δ* from TP75 and (F) *SRE2Δ* from TP8.
(TIF)

S6 Fig Multiplex PCR analysis of double mutants. Multiplex PCR analysis of the mutants shown in Fig 6B and 6C. The content of *mat1* was estimated by quantification of P- and M-specific band intensities. N.D. = Not-detected.
(TIF)

S1 Table List of strains used in crosses. (DOC)

S2 Table Bioneer mutants showing potentially biased mating-type ratios in fluorescence screen and their characterization by multiplex PCR for *mat1* content and by barcode sequencing. (XLSX)

S3 Table Bioneer mutants with altered iodine staining phenotypes and their characterization by multiplex PCR for *mat1* content and by barcode sequencing. (XLSX)

S4 Table Bioneer mutants excluded from the analysis. (XLSX)

## References

[1] Kelly M, Burke J, Smith M, Klar A, Beach D. Four mating-type genes control sexual differentiation in the fission yeast. EMBO J. 1988;7(5):1537–47. . 2900761 10.1002/j.1460-2075.1988.tb02973.x PMC458406

[2] Klar AJ, Ishikawa K, Moore S. A Unique DNA Recombination Mechanism of the Mating/Cell-type Switching of Fission Yeasts: a Review. Microbiol Spectr. 2014;2(5). doi: 10.1128/microbiolspec.MDNA3-0003-2014 . 26104357 10.1128/microbiolspec.MDNA3-0003-2014 PMC7687047

[3] Thon G, Klar AJ. Directionality of fission yeast mating-type interconversion is controlled by the location of the donor loci. Genetics. 1993;134(4):1045–54. . 8375648 10.1093/genetics/134.4.1045 PMC1205573

[4] Miyata H, Miyata M. Mode of Conjugation in Homothallic Cells of Schizosaccharomyces-Pombe. Journal of General and Applied Microbiology. 1981;27(5):365–71. doi: 10.2323/jgam.27.365

[5] Egel R, Eie B. Cell lineage asymmetry in Schizosaccharomyces pombe: unilateral transmission of a high-frequency state for mating-type switching in diploid pedigrees. Current Genetics. 1987;12(6):429–33. doi: 10.1007/bf00434820

[6] Dalgaard JZ, Klar AJ. Orientation of DNA replication establishes mating-type switching pattern in S. pombe. Nature. 1999;400(6740):181–4. doi: 10.1038/22139 . 10408447 10.1038/22139

[7] Dalgaard JZ, Klar AJS. swi1 and swi3 perform imprinting, pausing, and termination of DNA replication in S-pombe. Cell. 2000;102(6):745–51. doi: 10.1016/S0092-8674(00)00063-5 11030618 10.1016/s0092-8674(00)00063-5

[8] Klar AJ. Differentiated parental DNA strands confer developmental asymmetry on daughter cells in fission yeast. Nature. 1987;326(6112):466–70. doi: 10.1038/326466a0 . 3561486 10.1038/326466a0

[9] Klar AJS. The Developmental Fate of Fission Yeast-Cells Is Determined by the Pattern of Inheritance of Parental and Grandparental DNA Strands. Embo Journal. 1990;9(5):1407–15. 2328720 10.1002/j.1460-2075.1990.tb08256.x PMC551827

[10] Holmes AM, Kaykov A, Arcangioli B. Molecular and cellular dissection of mating-type switching steps in Schizosaccharomyces pombe. Mol Cell Biol. 2005;25(1):303–11. doi: 10.1128/MCB.25.1.303-311.2005 . 15601851 10.1128/MCB.25.1.303-311.2005 PMC538788

[11] Vengrova S, Dalgaard JZ. The wild-type Schizosaccharomyces pombe mat1 imprint consists of two ribonucleotides. EMBO Rep. 2006;7(1):59–65. doi: 10.1038/sj.embor.7400576 . 16299470 10.1038/sj.embor.7400576 PMC1369229

[12] Vengrova S, Dalgaard JZ. RNase-sensitive DNA modification(s) initiates S. pombe mating-type switching. Genes Dev. 2004;18(7):794–804. doi: 10.1101/gad.289404 . 15059961 10.1101/gad.289404 PMC387419

[13] Arcangioli B. A site- and strand-specific DNA break confers asymmetric switching potential in fission yeast. EMBO J. 1998;17(15):4503–10. doi: 10.1093/emboj/17.15.4503 . 9687516 10.1093/emboj/17.15.4503 PMC1170781

[14] Arcangioli B. Fate of mat1 DNA strands during mating-type switching in fission yeast. EMBO Rep. 2000;1(2):145–50. doi: 10.1093/embo-reports/kvd023 . 11265754 10.1093/embo-reports/kvd023 PMC1084255

[15] Kaykov A, Arcangioli B. A programmed strand-specific and modified nick in S. pombe constitutes a novel type of chromosomal imprint. Curr Biol. 2004;14(21):1924–8. doi: 10.1016/j.cub.2004.10.026 . 15530393 10.1016/j.cub.2004.10.026

[16] Zech J, Godfrey EL, Masai H, Hartsuiker E, Dalgaard JZ. The DNA-Binding Domain of S. pombe Mrc1 (Claspin) Acts to Enhance Stalling at Replication Barriers. PLoS One. 2015;10(7):e0132595 doi: 10.1371/journal.pone.0132595 . 26201080 10.1371/journal.pone.0132595 PMC4511789

[17] Holmes A, Roseaulin L, Schurra C, Waxin H, Lambert S, Zaratiegui M, et al Lsd1 and lsd2 control programmed replication fork pauses and imprinting in fission yeast. Cell Rep. 2012;2(6):1513–20. doi: 10.1016/j.celrep.2012.10.011 . 23260662 10.1016/j.celrep.2012.10.011 PMC3909218

[18] Raimondi C, Jagla B, Proux C, Waxin H, Gangloff S, Arcangioli B. Molecular signature of the imprintosome complex at the mating-type locus in fission yeast. Microb Cell. 2018;5(4):169–83. doi: 10.15698/mic2018.04.623 . 29610759 10.15698/mic2018.04.623 PMC5878685

[19] Kaykov A, Holmes AM, Arcangioli B. Formation, maintenance and consequences of the imprint at the mating-type locus in fission yeast. Embo Journal. 2004;23(4):930–8. doi: 10.1038/sj.emboj.7600099 14765111 10.1038/sj.emboj.7600099 PMC381007

[20] Yamada-Inagawa T, Klar AJ, Dalgaard JZ. Schizosaccharomyces pombe switches mating type by the synthesis-dependent strand-annealing mechanism. Genetics. 2007;177(1):255–65. doi: 10.1534/genetics.107.076315 . 17660548 10.1534/genetics.107.076315 PMC2013724

[21] Grewal SIS, Klar AJS. A recombinationally repressed region between mat2 and mat3 loci shares homology to centromeric repeats and regulates directionality of mating-type switching in fission yeast. Genetics. 1997;146(4):1221–38. 9258669 10.1093/genetics/146.4.1221 PMC1208070

[22] Jia S, Yamada T, Grewal SI. Heterochromatin regulates cell type-specific long-range chromatin interactions essential for directed recombination. Cell. 2004;119(4):469–80. doi: 10.1016/j.cell.2004.10.020 . 15537537 10.1016/j.cell.2004.10.020

[23] Jakociunas T, Holm LR, Verhein-Hansen J, Trusina A, Thon G. Two portable recombination enhancers direct donor choice in fission yeast heterochromatin. PLoS Genet. 2013;9(10):e1003762 doi: 10.1371/journal.pgen.1003762 . 24204285 10.1371/journal.pgen.1003762 PMC3812072

[24] Thon G, Hansen KR, Altes SP, Sidhu D, Singh G, Verhein-Hansen J, et al The Clr7 and Clr8 directionality factors and the Pcu4 cullin mediate heterochromatin formation in the fission yeast Schizosaccharomyces pombe. Genetics. 2005;171(4):1583–95. doi: 10.1534/genetics.105.048298 . 16157682 10.1534/genetics.105.048298 PMC1456086

[25] Thon G, Bjerling P, Bunner CM, Verhein-Hansen J. Expression-state boundaries in the mating-type region of fission yeast. Genetics. 2002;161(2):611–22. . 12072458 10.1093/genetics/161.2.611 PMC1462127

[26] Matsuda E, Sugioka-Sugiyama R, Mizuguchi T, Mehta S, Cui B, Grewal SI. A homolog of male sex-determining factor SRY cooperates with a transposon-derived CENP-B protein to control sex-specific directed recombination. Proc Natl Acad Sci U S A. 2011;108(46):18754–9. doi: 10.1073/pnas.1109988108 . 22042869 10.1073/pnas.1109988108 PMC3219157

[27] Akamatsu Y, Dziadkowiec D, Ikeguchi M, Shinagawa H, Iwasaki H. Two different Swi5-containing protein complexes are involved in mating-type switching and recombination repair in fission yeast. Proc Natl Acad Sci U S A. 2003;100(26):15770–5. doi: 10.1073/pnas.2632890100 . 14663140 10.1073/pnas.2632890100 PMC307643

[28] Akamatsu Y, Tsutsui Y, Morishita T, Siddique MS, Kurokawa Y, Ikeguchi M, et al Fission yeast Swi5/Sfr1 and Rhp55/Rhp57 differentially regulate Rhp51-dependent recombination outcomes. EMBO J. 2007;26(5):1352–62. doi: 10.1038/sj.emboj.7601582 . 17304215 10.1038/sj.emboj.7601582 PMC1817630

[29] Haruta N, Kurokawa Y, Murayama Y, Akamatsu Y, Unzai S, Tsutsui Y, et al The Swi5-Sfr1 complex stimulates Rhp51/Rad51- and Dmc1-mediated DNA strand exchange in vitro. Nat Struct Mol Biol. 2006;13(9):823–30. doi: 10.1038/nsmb1136 . 16921379 10.1038/nsmb1136

[30] Kurokawa Y, Murayama Y, Haruta-Takahashi N, Urabe I, Iwasaki H. Reconstitution of DNA strand exchange mediated by Rhp51 recombinase and two mediators. PLoS Biol. 2008;6(4):e88 doi: 10.1371/journal.pbio.0060088 . 18416603 10.1371/journal.pbio.0060088 PMC2292753

[31] Yu C, Bonaduce MJ, Klar AJ. Going in the right direction: mating-type switching of Schizosaccharomyces pombe is controlled by judicious expression of two different swi2 transcripts. Genetics. 2012;190(3):977–87. doi: 10.1534/genetics.111.137109 . 22209903 10.1534/genetics.111.137109 PMC3296259

[32] Rudolph C, Kunz C, Parisi S, Lehmann E, Hartsuiker E, Fartmann B, et al The msh2 gene of Schizosaccharomyces pombe is involved in mismatch repair, mating-type switching, and meiotic chromosome organization. Molecular and Cellular Biology. 1999;19(1):241–50. 9858548 10.1128/mcb.19.1.241 PMC83882

[33] Tsutsui Y, Khasanov FK, Shinagawa H, Iwasaki H, Bashkirov VI. Multiple interactions among the components of the recombinational DNA repair system in Schizosaccharomyces pombe. Genetics. 2001;159(1):91–105. 11560889 10.1093/genetics/159.1.91 PMC1461803

[34] Fleck O, Rudolph C, Albrecht A, Lorentz A, Schar P, Schmidt H. The mutator gene swi8 effects specific mutations in the mating-type region of Schizosaccharomyces pombe. Genetics. 1994;138(3):621–32. . 7851760 10.1093/genetics/138.3.621 PMC1206213

[35] Vagin DA, Khasanov FK, Bashkirov VI. [The role of recombinational repair proteins in mating type switching in fission yeast cells]. Genetika. 2006;42(4):487–93. . 16756067

[36] Roseaulin L, Yamada Y, Tsutsui Y, Russell P, Iwasaki H, Arcangioli B. Mus81 is essential for sister chromatid recombination at broken replication forks. Embo Journal. 2008;27(9):1378–87. doi: 10.1038/emboj.2008.65 18388861 10.1038/emboj.2008.65 PMC2374842

[37] Schmidt H, Kapitzafecke P, Stephen ER, Gutz H. Some of the Swi Genes of Schizosaccharomyces-Pombe Also Have a Function in the Repair of Radiation-Damage. Current Genetics. 1989;16(2):89–94. doi: 10.1007/Bf00393400 2598273 10.1007/BF00393400

[38] Ostermann K, Lorentz A, Schmidt H. The Fission Yeast Rad22 Gene, Having a Function in Mating-Type Switching and Repair of DNA Damages, Encodes a Protein Homolog to Rad52 of Saccharomyces-Cerevisiae. Nucleic Acids Research. 1993;21(25):5940–4. doi: 10.1093/nar/21.25.5940 8290356 10.1093/nar/21.25.5940 PMC310478

[39] Zhang JM, Liu XM, Ding YH, Xiong LY, Ren JY, Zhou ZX, et al Fission yeast Pxd1 promotes proper DNA repair by activating Rad16XPF and inhibiting Dna2. PLoS Biol. 2014;12(9):e1001946 doi: 10.1371/journal.pbio.1001946 . 25203555 10.1371/journal.pbio.1001946 PMC4159138

[40] Egel R, Beach DH, Klar AJ. Genes required for initiation and resolution steps of mating-type switching in fission yeast. Proc Natl Acad Sci U S A. 1984;81(11):3481–5. . 6587363 10.1073/pnas.81.11.3481 PMC345532

[41] Arcangioli B, Klar AJ. A novel switch-activating site (SAS1) and its cognate binding factor (SAP1) required for efficient mat1 switching in Schizosaccharomyces pombe. EMBO J. 1991;10(10):3025–32. . 1915277 10.1002/j.1460-2075.1991.tb07853.x PMC453017

[42] Thon G, Klar AJ. The clr1 locus regulates the expression of the cryptic mating-type loci of fission yeast. Genetics. 1992;131(2):287–96. . 1644273 10.1093/genetics/131.2.287 PMC1205004

[43] Ekwall K, Ruusala T. Mutations in rik1, clr2, clr3 and clr4 genes asymmetrically derepress the silent mating-type loci in fission yeast. Genetics. 1994;136(1):53–64. . 8138176 10.1093/genetics/136.1.53 PMC1205792

[44] Singh J, Goel V, Klar AJ. A novel function of the DNA repair gene rhp6 in mating-type silencing by chromatin remodeling in fission yeast. Mol Cell Biol. 1998;18(9):5511–22. . 9710635 10.1128/mcb.18.9.5511 PMC109136

[45] Nonaka N, Kitajima T, Yokobayashi S, Xiao G, Yamamoto M, Grewal SI, et al Recruitment of cohesin to heterochromatic regions by Swi6/HP1 in fission yeast. Nat Cell Biol. 2002;4(1):89–93. doi: 10.1038/ncb739 . 11780129 10.1038/ncb739

[46] Shankaranarayana GD, Motamedi MR, Moazed D, Grewal SIS. Sir2 Regulates Histone H3 Lysine 9 Methylation and Heterochromatin Assembly in Fission Yeast. Current Biology. 2003;13(14):1240–6. doi: 10.1016/s0960-9822(03)00489-5 12867036 10.1016/s0960-9822(03)00489-5

[47] Jia S, Kobayashi R, Grewal SI. Ubiquitin ligase component Cul4 associates with Clr4 histone methyltransferase to assemble heterochromatin. Nat Cell Biol. 2005;7(10):1007–13. doi: 10.1038/ncb1300 . 16127433 10.1038/ncb1300

[48] Aguilar-Arnal L, Marsellach FX, Azorin F. The fission yeast homologue of CENP-B, Abp1, regulates directionality of mating-type switching. EMBO J. 2008;27(7):1029–38. doi: 10.1038/emboj.2008.53 . 18354497 10.1038/emboj.2008.53 PMC2323252

[49] Lawrence RJ, Volpe TA. Msc1 links dynamic Swi6/HP1 binding to cell fate determination. Proc Natl Acad Sci U S A. 2009;106(4):1163–8. doi: 10.1073/pnas.0811161106 . 19164572 10.1073/pnas.0811161106 PMC2633577

[50] Dubey RN, Nakwal N, Bisht KK, Saini A, Haldar S, Singh J. Interaction of APC/C-E3 ligase with Swi6/HP1 and Clr4/Suv39 in heterochromatin assembly in fission yeast. J Biol Chem. 2009;284(11):7165–76. doi: 10.1074/jbc.M806461200 . 19117951 10.1074/jbc.M806461200 PMC2652303

[51] Lorentz A, Heim L, Schmidt H. The Switching Gene Swi6 Affects Recombination and Gene-Expression in the Mating-Type Region of Schizosaccharomyces Pombe. Molecular & General Genetics. 1992;233(3):436–42. 1620099 10.1007/BF00265441

[52] Li F, Goto DB, Zaratiegui M, Tang X, Martienssen R, Cande WZ. Two novel proteins, dos1 and dos2, interact with rik1 to regulate heterochromatic RNA interference and histone modification. Curr Biol. 2005;15(16):1448–57. doi: 10.1016/j.cub.2005.07.021 . 16040243 10.1016/j.cub.2005.07.021

[53] Horn PJ, Bastie JN, Peterson CL. A Rik1-associated, cullin-dependent E3 ubiquitin ligase is essential for heterochromatin formation. Genes & Development. 2005;19(14):1705–14. doi: 10.1101/gad.1328005 16024659 10.1101/gad.1328005 PMC1176008

[54] Hong E-JE, Villén J, Moazed D. A Cullin E3 Ubiquitin Ligase Complex Associates with Rik1 and the Clr4 Histone H3-K9 Methyltransferase and is Required for RNAi-Mediated Heterochromatin Formation. RNA Biology. 2005;2(3):106–11. doi: 10.4161/rna.2.3.2131 17114925 10.4161/rna.2.3.2131

[55] Zhang K, Mosch K, Fischle W, Grewal SI. Roles of the Clr4 methyltransferase complex in nucleation, spreading and maintenance of heterochromatin. Nat Struct Mol Biol. 2008;15(4):381–8. doi: 10.1038/nsmb.1406 . 18345014 10.1038/nsmb.1406

[56] Alper BJ, Job G, Yadav RK, Shanker S, Lowe BR, Partridge JF. Sir2 is required for Clr4 to initiate centromeric heterochromatin assembly in fission yeast. Embo Journal. 2013;32(17):2321–35. doi: 10.1038/emboj.2013.143 23771057 10.1038/emboj.2013.143 PMC3770337

[57] Buscaino A, Lejeune E, Audergon P, Hamilton G, Pidoux A, Allshire RC. Distinct roles for Sir2 and RNAi in centromeric heterochromatin nucleation, spreading and maintenance. EMBO J. 2013;32(9):1250–64. doi: 10.1038/emboj.2013.72 . 23572080 10.1038/emboj.2013.72 PMC3642681

[58] Freeman-Cook LL, Gomez EB, Spedale EJ, Marlett J, Forsburg SL, Pillus L, et al Conserved locus-specific silencing functions of Schizosaccharomyces pombe sir2+. Genetics. 2005;169(3):1243–60. doi: 10.1534/genetics.104.032714 . 15545655 10.1534/genetics.104.032714 PMC1449530

[59] Xhemalce B, Kouzarides T. A chromodomain switch mediated by histone H3 Lys 4 acetylation regulates heterochromatin assembly. Genes Dev. 2010;24(7):647–52. doi: 10.1101/gad.1881710 . 20299449 10.1101/gad.1881710 PMC2849121

[60] Noma K, Grewal SIS. Histone H3 lysine 4 methylation is mediated by Set1 and promotes maintenance of active chromatin states in fission yeast. Proceedings of the National Academy of Sciences of the United States of America. 2002;99:16438–45. doi: 10.1073/pnas.182436399 12193658 10.1073/pnas.182436399 PMC139906

[61] Roguev A, Schaft D, Shevchenko A, Aasland R, Shevchenko A, Stewart AF. High conservation of the Set1/Rad6 axis of histone 3 lysine 4 methylation in budding and fission yeasts. J Biol Chem. 2003;278(10):8487–93. doi: 10.1074/jbc.M209562200 . 12488447 10.1074/jbc.M209562200

[62] Sugiyama T, Cam HP, Sugiyama R, Noma K, Zofall M, Kobayashi R, et al SHREC, an effector complex for heterochromatic transcriptional silencing. Cell. 2007;128(3):491–504. doi: 10.1016/j.cell.2006.12.035 . 17289569 10.1016/j.cell.2006.12.035

[63] Yamada T, Fischle W, Sugiyama T, Allis CD, Grewal SI. The nucleation and maintenance of heterochromatin by a histone deacetylase in fission yeast. Mol Cell. 2005;20(2):173–85. doi: 10.1016/j.molcel.2005.10.002 . 16246721 10.1016/j.molcel.2005.10.002

[64] Cam HP, Noma K, Ebina H, Levin HL, Grewal SI. Host genome surveillance for retrotransposons by transposon-derived proteins. Nature. 2008;451(7177):431–6. doi: 10.1038/nature06499 . 18094683 10.1038/nature06499

[65] Hall IM, Shankaranarayana GD, Noma KI, Ayoub N, Cohen A, Grewal SIS. Establishment and maintenance of a heterochromatin domain. Science. 2002;297(5590):2232–7. doi: 10.1126/science.1076466 12215653 10.1126/science.1076466

[66] Kim HS, Choi ES, Shin JA, Jang YK, Park SD. Regulation of Swi6/HP1-dependent heterochromatin assembly by cooperation of components of the mitogen-activated protein kinase pathway and a histone deacetylase Clr6. J Biol Chem. 2004;279(41):42850–9. doi: 10.1074/jbc.M407259200 . 15292231 10.1074/jbc.M407259200

[67] Hansen KR, Hazan I, Shanker S, Watt S, Verhein-Hansen J, Bahler J, et al H3K9me-independent gene silencing in fission yeast heterochromatin by Clr5 and histone deacetylases. PLoS Genet. 2011;7(1):e1001268 doi: 10.1371/journal.pgen.1001268 . 21253571 10.1371/journal.pgen.1001268 PMC3017117

[68] Shimada A, Dohke K, Sadaie M, Shinmyozu K, Nakayama J, Urano T, et al Phosphorylation of Swi6/HP1 regulates transcriptional gene silencing at heterochromatin. Genes Dev. 2009;23(1):18–23. doi: 10.1101/gad.1708009 . 19136623 10.1101/gad.1708009 PMC2632169

[69] Szklarczyk D, Franceschini A, Wyder S, Forslund K, Heller D, Huerta-Cepas J, et al STRING v10: protein-protein interaction networks, integrated over the tree of life. Nucleic Acids Res. 2015;43(Database issue):D447–52. doi: 10.1093/nar/gku1003 . 25352553 10.1093/nar/gku1003 PMC4383874

[70] Jahn LJ, Mason B, Brogger P, Toteva T, Nielsen DK, Thon G. Dependency of Heterochromatin Domains on Replication Factors. G3 (Bethesda). 2017 doi: 10.1534/g3.117.300341 . 29187422 10.1534/g3.117.300341 PMC5919735

[71] Mamnun YM, Katayama S, Toda T. Fission yeast Mcl1 interacts with SCFPof3 and is required for centromere formation. Biochemical and Biophysical Research Communications. 2006;350(1):125–30. doi: 10.1016/j.bbrc.2006.09.024 16997270 10.1016/j.bbrc.2006.09.024

[72] Katayama S, Kitamura K, Lehmann A, Nikaido O, Toda T. Fission yeast F-box protein Pof3 is required for genome integrity and telomere function. Mol Biol Cell. 2002;13(1):211–24. doi: 10.1091/mbc.01-07-0333 . 11809834 10.1091/mbc.01-07-0333 PMC65083

[73] Otero G, Fellows J, Li Y, de Bizemont T, Dirac AMG, Gustafsson CM, et al Elongator, a multisubunit component of a novel RNA polymerase II holoenzyme for transcriptional elongation. Molecular Cell. 1999;3(1):109–18. doi: 10.1016/S1097-2765(00)80179-3 10024884 10.1016/s1097-2765(00)80179-3

[74] Krogan NJ, Greenblatt JF. Characterization of a six-subunit holo-elongator complex required for the regulated expression of a group of genes in Saccharomyces cerevisiae. Mol Cell Biol. 2001;21(23):8203–12. doi: 10.1128/MCB.21.23.8203-8212.2001 . 11689709 10.1128/MCB.21.23.8203-8212.2001 PMC99985

[75] Wittschieben BO, Otero G, de Bizemont T, Fellows J, Erdjument-Bromage H, Ohba R, et al A novel histone acetyltransferase is an integral subunit of elongating RNA polymerase II holoenzyme. Molecular Cell. 1999;4(1):123–8. doi: 10.1016/S1097-2765(00)80194-X 10445034 10.1016/s1097-2765(00)80194-x

[76] Esberg A, Huang B, Johansson MJ, Bystrom AS. Elevated levels of two tRNA species bypass the requirement for elongator complex in transcription and exocytosis. Mol Cell. 2006;24(1):139–48. doi: 10.1016/j.molcel.2006.07.031 . 17018299 10.1016/j.molcel.2006.07.031

[77] Glatt S, Seraphin B, Muller CW. Elongator: transcriptional or translational regulator? Transcription. 2012;3(6):273–6. doi: 10.4161/trns.21525 . 22889844 10.4161/trns.21525 PMC3630180

[78] Mikheyeva IV, Grady PJ, Tamburini FB, Lorenz DR, Cam HP. Multifaceted genome control by Set1 Dependent and Independent of H3K4 methylation and the Set1C/COMPASS complex. PLoS Genet. 2014;10(10):e1004740 doi: 10.1371/journal.pgen.1004740 . 25356590 10.1371/journal.pgen.1004740 PMC4214589

[79] Morohashi H, Maculins T, Labib K. The Amino-Terminal TPR Domain of Dia2 Tethers SCFDia2 to the Replisome Progression Complex. Current Biology. 2009;19(22):1943–9. doi: 10.1016/j.cub.2009.09.062 19913425 10.1016/j.cub.2009.09.062

[80] Roseaulin LC, Noguchi C, Martinez E, Ziegler MA, Toda T, Noguchi E. Coordinated Degradation of Replisome Components Ensures Genome Stability upon Replication Stress in the Absence of the Replication Fork Protection Complex. Plos Genetics. 2013;9(1). doi: 10.1371/journal.pgen.1003213 23349636 10.1371/journal.pgen.1003213 PMC3547854

[81] Takayama Y, Mamnun YM, Trickey M, Dhut S, Masuda F, Yamano H, et al Hsk1-and SCFPof3-Dependent Proteolysis of S. pombe Ams2 Ensures Histone Homeostasis and Centromere Function. Developmental Cell. 2010;18(3):385–96. doi: 10.1016/j.devcel.2009.12.024 20230746 10.1016/j.devcel.2009.12.024 PMC2880248

[82] Kim KD, Tanizawa H, Iwasaki O, Noma K. Transcription factors mediate condensin recruitment and global chromosomal organization in fission yeast. Nat Genet. 2016;48(10):1242–52. doi: 10.1038/ng.3647 . 27548313 10.1038/ng.3647 PMC5042855

[83] Gonzalez M, He H, Sun S, Li C, Li F. Cell cycle-dependent deposition of CENP-A requires the Dos1/2-Cdc20 complex. Proc Natl Acad Sci U S A. 2013;110(2):606–11. doi: 10.1073/pnas.1214874110 . 23267073 10.1073/pnas.1214874110 PMC3545758

[84] Chen ES, Zhang K, Nicolas E, Cam HP, Zofall M, Grewal SI. Cell cycle control of centromeric repeat transcription and heterochromatin assembly. Nature. 2008;451(7179):734–7. doi: 10.1038/nature06561 . 18216783 10.1038/nature06561

[85] Kloc A, Zaratiegui M, Nora E, Martienssen R. RNA interference guides histone modification during the S phase of chromosomal replication. Curr Biol. 2008;18(7):490–5. doi: 10.1016/j.cub.2008.03.016 . 18394897 10.1016/j.cub.2008.03.016 PMC2408823

[86] Bar-Ziv R, Voichek Y, Barkai N. Chromatin dynamics during DNA replication. Genome Res. 2016;26(9):1245–56. doi: 10.1101/gr.201244.115 . 27225843 10.1101/gr.201244.115 PMC5052047

[87] Abshiru N, Rajan RE, Verreault A, Thibault P. Unraveling Site-Specific and Combinatorial Histone Modifications Using High-Resolution Mass Spectrometry in Histone Deacetylase Mutants of Fission Yeast. J Proteome Res. 2016;15(7):2132–42. doi: 10.1021/acs.jproteome.5b01156 . 27223649 10.1021/acs.jproteome.5b01156

[88] Sollier J, Lin WK, Soustelle C, Suhre K, Nicolas A, Geli V, et al Set1 is required for meiotic S-phase onset, double-strand break formation and middle gene expression. Embo Journal. 2004;23(9):1957–67. doi: 10.1038/sj.emboj.7600204 15071505 10.1038/sj.emboj.7600204 PMC404324

[89] Sommermeyer V, Beneut C, Chaplais E, Serrentino ME, Borde V. Spp1, a member of the Set1 Complex, promotes meiotic DSB formation in promoters by tethering histone H3K4 methylation sites to chromosome axes. Mol Cell. 2013;49(1):43–54. doi: 10.1016/j.molcel.2012.11.008 . 23246437 10.1016/j.molcel.2012.11.008

[90] Tischfield SE, Keeney S. Scale matters The spatial correlation of yeast meiotic DNA breaks with histone H3 trimethylation is driven largely by independent colocalization at promoters. Cell Cycle. 2012;11(8):1496–503. doi: 10.4161/cc.19733 22433953 10.4161/cc.19733 PMC3341227

[91] Zofall M, Grewal SIS. HULC, a histone H2B ubiquitinating complex, modulates heterochromatin independent of histone methylation in fission yeast. Journal of Biological Chemistry. 2007;282(19):14065–72. doi: 10.1074/jbc.M700292200 17363370 10.1074/jbc.M700292200

[92] Tanny JC, Erdjument-Bromage H, Tempst P, Allis CD. Ubiquitylation of histone H2B controls RNA polymerase II transcription elongation independently of histone H3 methylation. Genes Dev. 2007;21(7):835–47. doi: 10.1101/gad.1516207 . 17374714 10.1101/gad.1516207 PMC1838534

[93] Racine A, Page V, Nagy S, Grabowski D, Tanny JC. Histone H2B ubiquitylation promotes activity of the intact Set1 histone methyltransferase complex in fission yeast. J Biol Chem. 2012;287(23):19040–7. doi: 10.1074/jbc.M112.356253 . 22505722 10.1074/jbc.M112.356253 PMC3365937

[94] Pebernard S, Wohlschlegel J, McDonald WH, Yates JR 3rd, Boddy MN. The Nse5-Nse6 dimer mediates DNA repair roles of the Smc5-Smc6 complex. Mol Cell Biol. 2006;26(5):1617–30. doi: 10.1128/MCB.26.5.1617-1630.2006 . 16478984 10.1128/MCB.26.5.1617-1630.2006 PMC1430260

[95] Prudden J, Pebernard S, Raffa G, Slavin DA, Perry JJP, Tainer JA, et al SUMO-targeted ubiquitin ligases in genome stability. Embo Journal. 2007;26(18):4089–101. doi: 10.1038/sj.emboj.7601838 17762865 10.1038/sj.emboj.7601838 PMC2230673

[96] Ekwall K, Thon G. Setting up Schizosaccharomyces pombe Crosses/Matings. Cold Spring Harb Protoc. 2017;2017(7):pdb prot091694. doi: 10.1101/pdb.prot091694 . 28679701 10.1101/pdb.prot091694

[97] Roguev A, Xu J, Krogan N. DNA Preparation from Schizosaccharomyces pombe. Cold Spring Harb Protoc. 2017 doi: 10.1101/pdb.prot091959 . 28733411 10.1101/pdb.prot091959

[98] Shigemori Y, Mikawa T, Shibata T, Oishi M. Multiplex PCR: use of heat-stable Thermus thermophilus RecA protein to minimize non-specific PCR products. Nucleic Acids Res. 2005;33(14):e126 doi: 10.1093/nar/gni111 . 16087733 10.1093/nar/gni111 PMC1183492

