# Supplementary figures and images for: New insights into donor directionality of mating-type switching in Schizosaccharomyces pombe
Source: PLoS Genet. 2018 May 31;14(5):e1007424. doi: 10.1371/journal.pgen.1007424 (PMC6007933; doi:10.1371/journal.pgen.1007424)

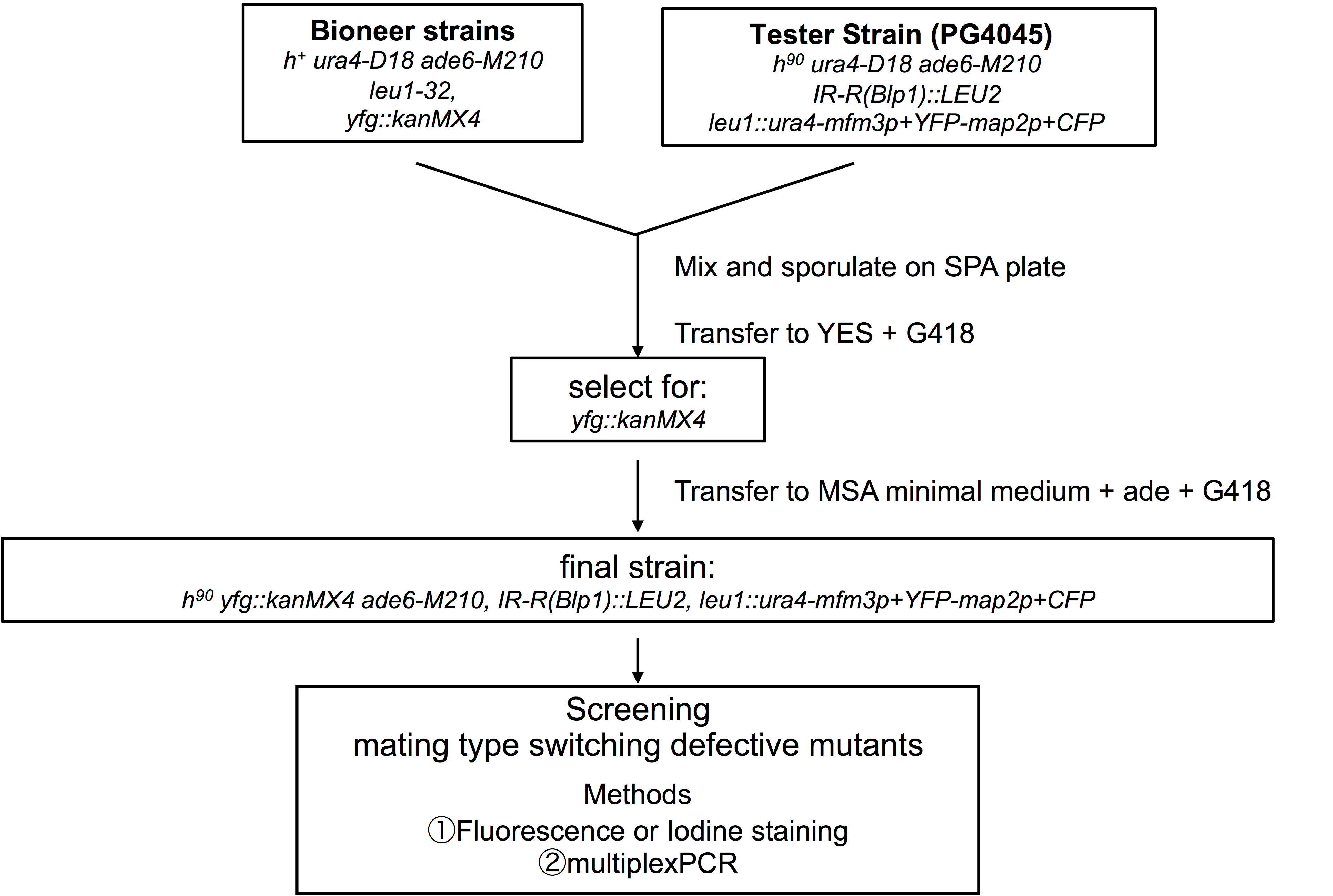

Supplement: S1 Fig — The Bioneer collection of S. pombe haploid deletion strains (V5) was mated with PG4045 on SPA plates in an arrayed format using a high-throughput robot (Singer Inst.). Spores were selected, germinated, and G418-resistant progeny that were also prototrophic for leucine (selecting for h90 mating-type region) and uracil (selecting for cell-type specific fluorescent reporters) were obtained, colony purified, re-arrayed, and examined by a combination of fluorescence microscopy with a high throughput Opera microscope (all strains), iodine staining of colonies (subset of strains with unclear fluorescence output), and multiplex PCR (subset of strains with potential mating-type switching defects obtained in the first screens). (TIF) [file pgen.1007424.s001.tif]

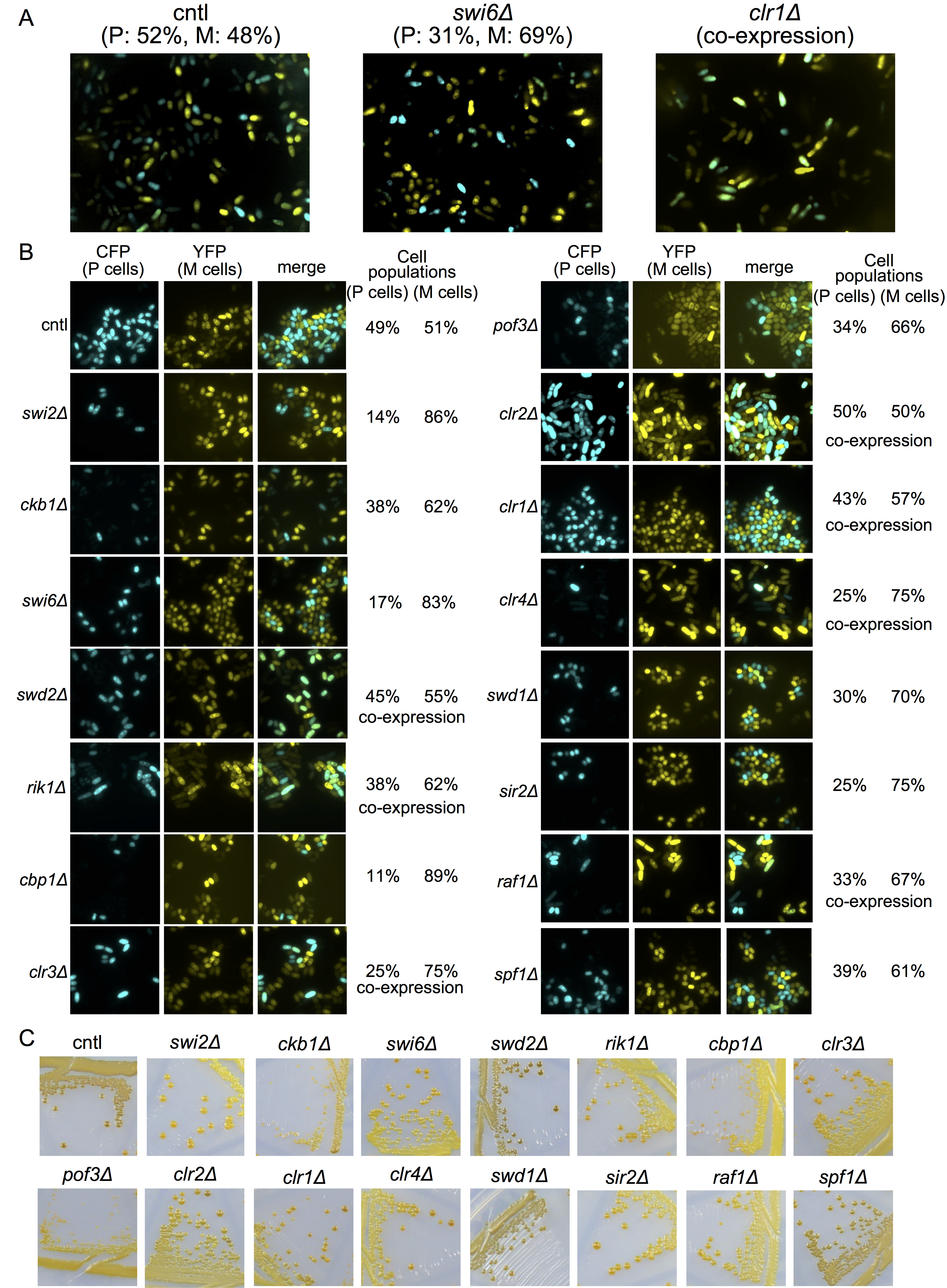

Supplement: S2 Fig — (A) Merged channels for wild-type h90 strain (cntl) and two mutants. The 15 mutants shown in (B, C) were identified by fluorescence microscopy or iodine staining analysis in the screen outlined in S1 Fig. (B) Repeat of cell-type quantification using a DeltaVision Elite microscope (GE Healthcare). (C) Iodine staining of strains with the indicated gene deletions. (TIF) [file pgen.1007424.s002.tif]

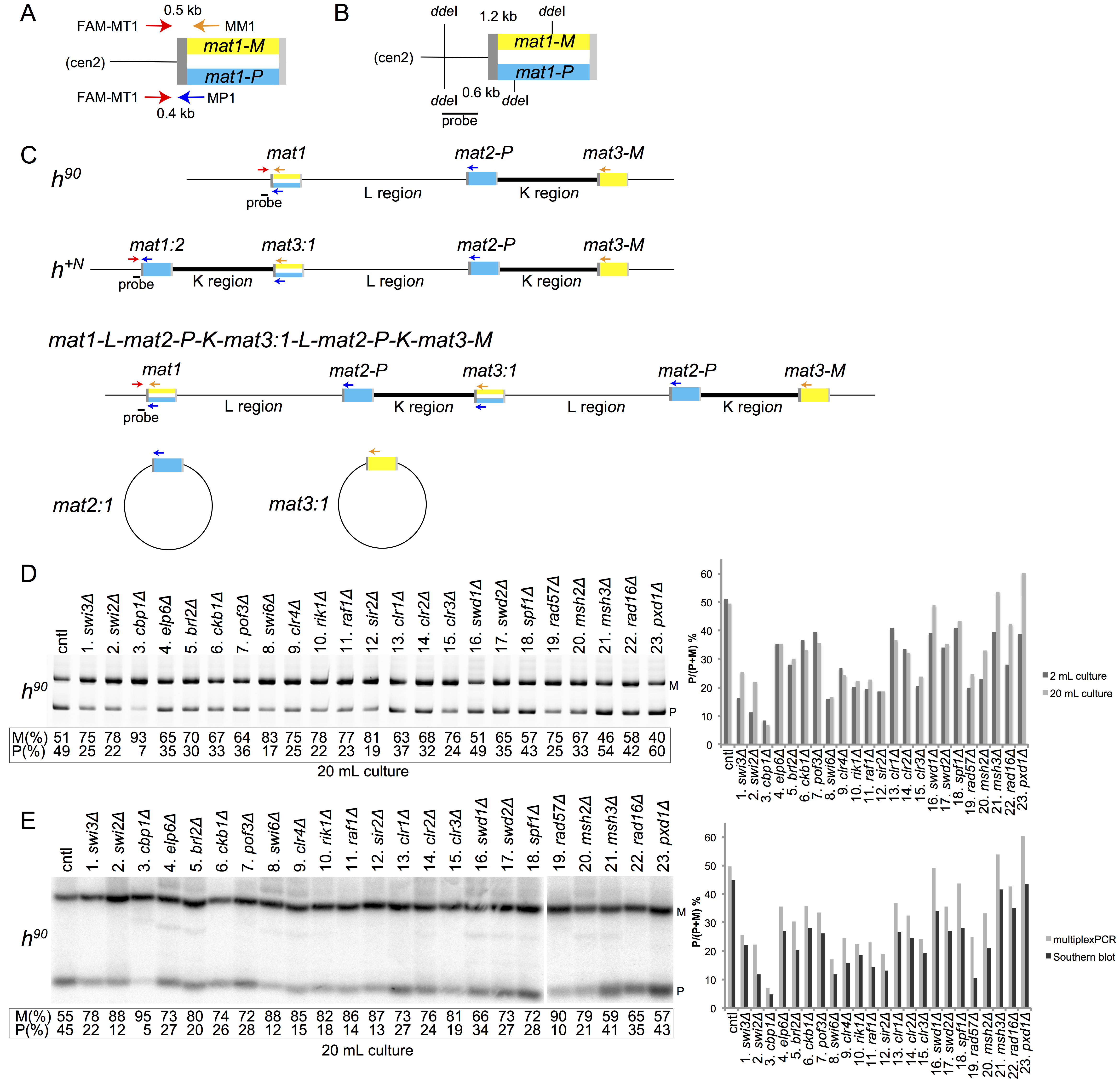

Supplement: S3 Fig — (A, B) Representation of the mat1 region showing (A) priming sites used for multiplex PCR and (B) the restriction sites and probe (DdeI-NsiI fragment) used for the Southern blots in (E). (C) Location of primers and probe complementarity on h90, h+N, duplicated mating-type region (mat1-L-mat2-P-K-mat3:1-L-mat2-P-K-mat3-M), and mat2:1 or mat3:1 circles. (D) 30 μL of saturated YES pre-cultures (2 ml each) were used to inoculate 20 mL YES cultures that were then propagated until late exponential phase. DNA was extracted from both the 2 ml pre-cultures and the 20 ml cultures and analyzed by multiplex PCR. (E) DNA preps from 20 mL YES cultures were analyzed by Southern blot taking advantage of the size difference between a mat1-P and mat1-M fragment in DdeI digests. Measurements by multiplex PCR and Southern blots are compared. (TIF) [file pgen.1007424.s003.tif]

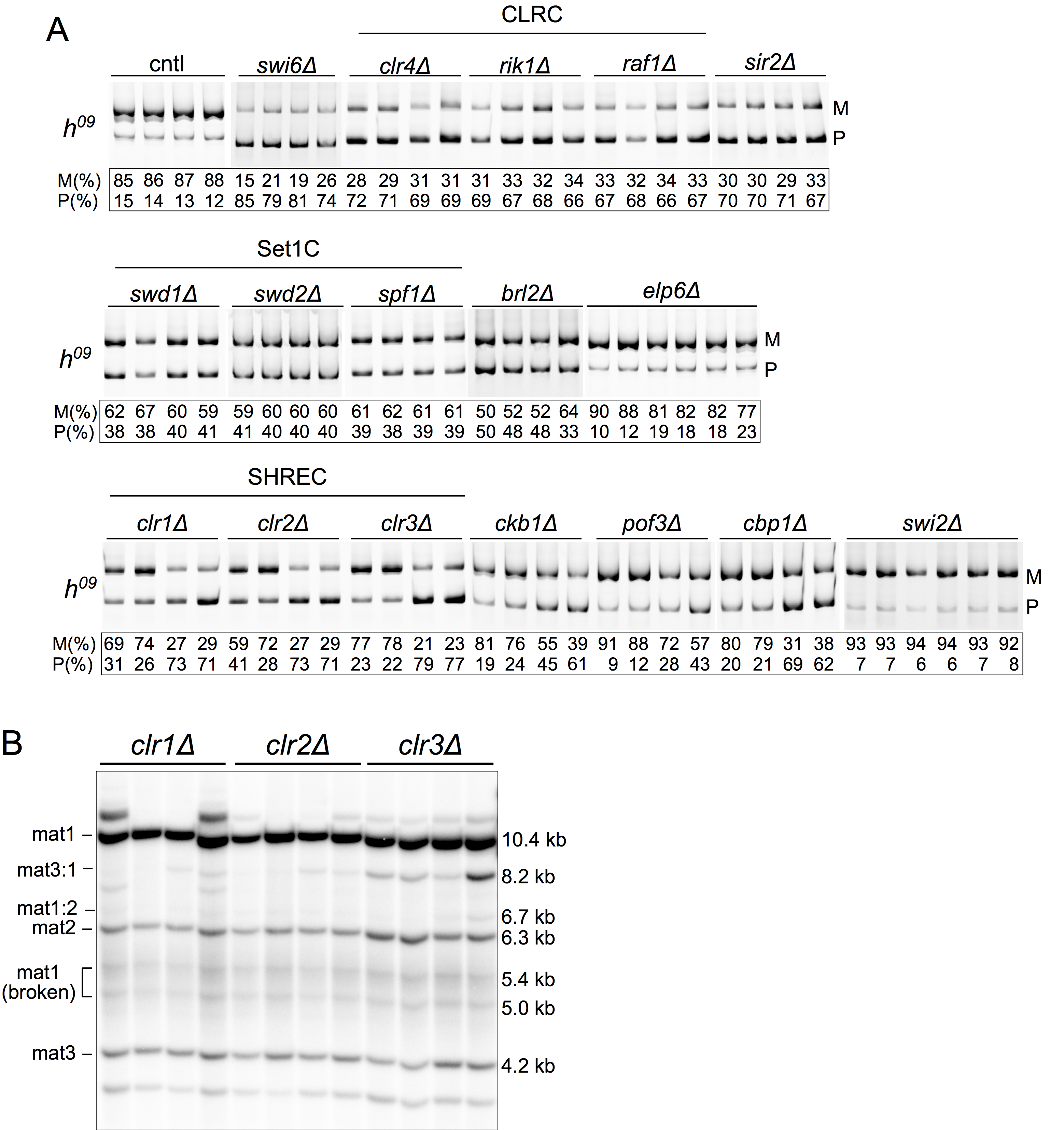

Supplement: S4 Fig — (A) Multiplex PCR analysis of the mutants shown in Fig 4. The content of mat1 was estimated by quantification of P- and M-specific band intensities. (B) Southern blot analysis using HindIII digests of genomic DNA and a 10.4 kb mat1 HindIII fragment as probe. (TIF) [file pgen.1007424.s004.tif]

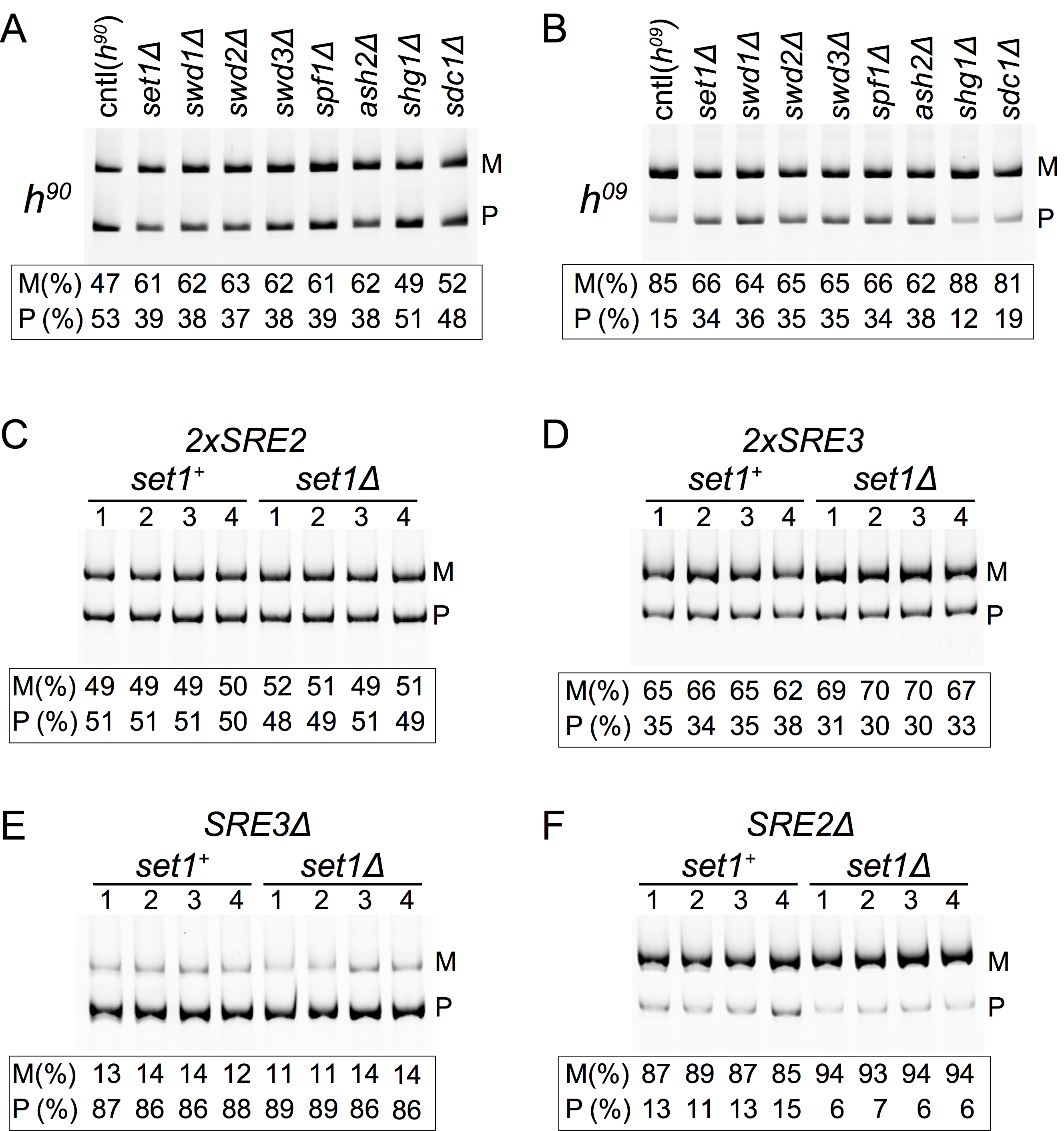

Supplement: S5 Fig — (A, B) Multiplex PCR analysis of the mutants shown in Fig 5C and 5D and quantification of mat1 content estimated from P- and M-specific band intensities. The relative P band intensity in each lane (P/(P+M)) was calculated from (A) h90 and (B) h09. (C-F) Epistasis analysis by multiplex PCR analysis for the Set1 and SRE elements mutants shown in Fig 5E–5H. The relative P band intensity (P/(P+M)) was calculated for each lane. (C) 2×SRE2 (mat2-P-SRE2 mat3-M-SRE2) strains were derived from strain TP126; (D) 2×SRE3 (mat2-P-SRE3 mat3-M-SRE3) strains were derived from strain TP303; (E) SRE3Δ from TP75 and (F) SRE2Δ from TP8. (TIF) [file pgen.1007424.s005.tif]

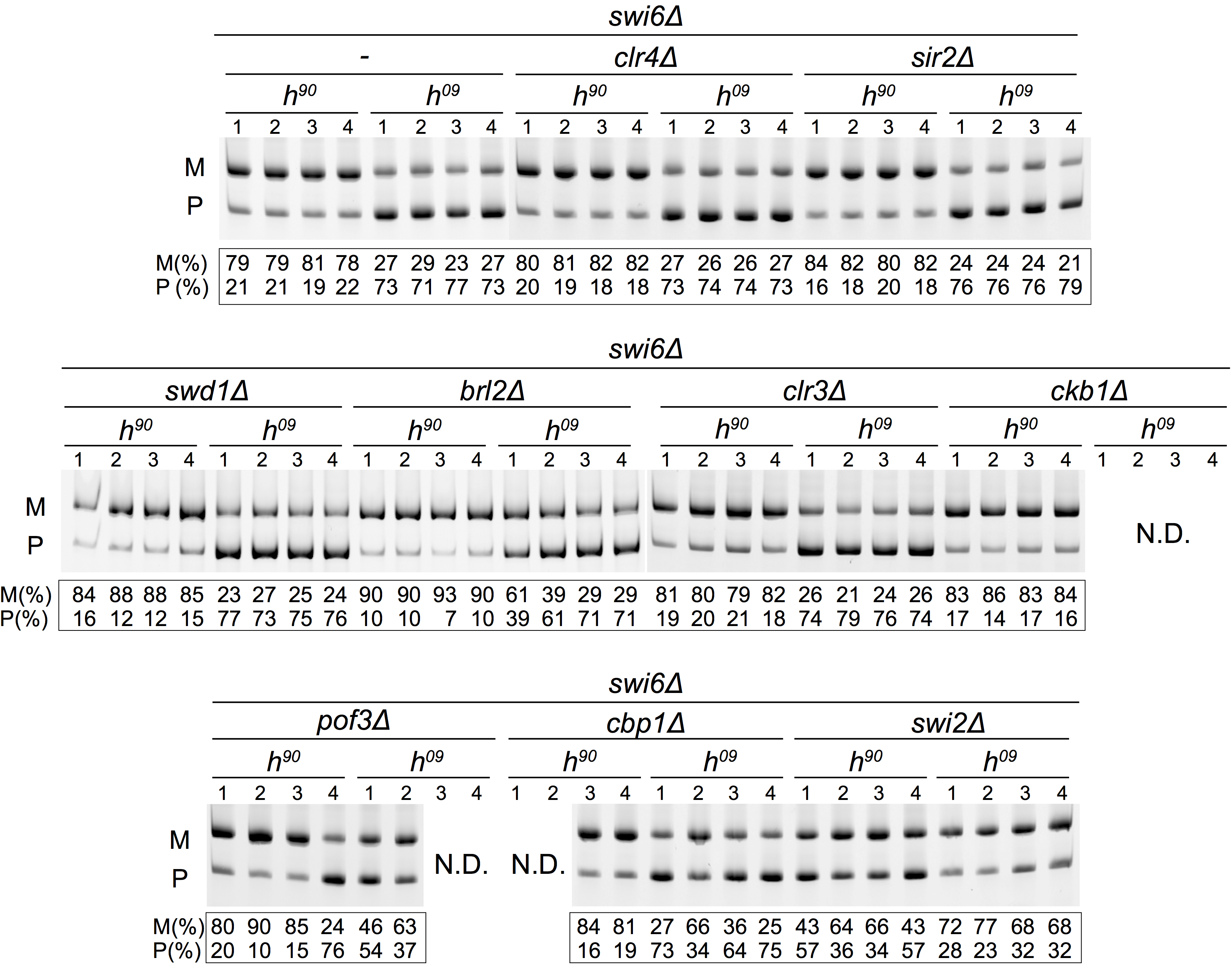

Supplement: S6 Fig — Multiplex PCR analysis of the mutants shown in Fig 6B and 6C. The content of mat1 was estimated by quantification of P- and M-specific band intensities. N.D. = Not-detected. (TIF) [file pgen.1007424.s006.tif]
