# Supplementary material for: New insights into donor directionality of mating-type switching in Schizosaccharomyces pombe
Source: PLoS Genet. 2018 May 31;14(5):e1007424. doi: 10.1371/journal.pgen.1007424 (PMC6007933; doi:10.1371/journal.pgen.1007424)
Supplement: S1 Table — (DOC) [file pgen.1007424.s007.doc]

**Table S1. List of strains used in crosses.**

| **Strain** | **Mating-type region** | **Other** |
| --- | --- | --- |
| **Bioneer** | *h^+N^* | *yfg::kan ura4-D18 leu1-32 ade6-210/216* |
| **PG4045** | *h^90^* | *ura4-D18 leu1::ura4-mfm3p+YFP-map2p+CFP ade6-216 (Blp1)::LEU2* |
| **PG4048** | *h^09^* | *ura4-D18 leu1::ura4-mfm3p+YFP-map2p+CFP ade6-216 (Blp1)::LEU2* |
| **TM277** | *h^+N^* | *swd2::kan ura4-D18 leu1-32* |
| **P1-1G** | *h^+^* | *sdc1::kan ura4-D18 leu1-32 ade6-M21?* |
| **JK499** | *h^+^* | *set1::kan ura4-D18 leu1-32* |
| **TM360** | *h^90^* | *swi6Δ::ura4 ura4-D18 leu1-32 ade6-216 (Blp1)::LEU2* |
| **TM361** | *h^09^* | *swi6Δ::ura4 ura4-D18 leu1-32 ade6-216 (Blp1)::LEU2* |
| **TP8** | *mat2-PΔSRE2* | *ura4-D18 leu1-32 ade6-210* |
| **TP75** | *mat3-MΔSRE3* | *ura4-D18 leu1-32 ade6-216* |
| **TP126** | *mat3-M-SRE2* | *ura4-D18 leu1-32 ade6-216* |
| **TP303** | *mat2-P-SRE3* | *ura4-D18 leu1-32 ade6-210* |
